# Physiological responses and transcriptomic analysis of *StCPD* gene overexpression in potato under salt stresses

**DOI:** 10.3389/fpls.2024.1297812

**Published:** 2024-02-16

**Authors:** Xiangyan Zhou, Yanming Ma, Rong Miao, Caijuan Li, Ziliang Liu, Dan Zhang, Sijin Chen, Jiaqi Luo, Wenhui Tang

**Affiliations:** ^1^ State Key Laboratory of Aridland Crop Science/College of Life Science and Technology, Gansu Agricultural University, Lanzhou, China; ^2^ Qinzhou District Agricultural Technology Comprehensive Service Center in Tianshui City, Tianshui, China; ^3^ Zhuanglang Agricultural Technology Extension Center in Pingliang City, Pingliang, China

**Keywords:** potato, *StCPD* gene, salt stress, transcriptome, brassinosteroids metabolism

## Abstract

**Introduction:**

The potato (*Solanum tuberosum* L.), one of the most vital food crops worldwide, is sensitive to salinity. Brassinosteroids (BRs) are crucial in tolerance to various abiotic stresses. The constitutive photomorphogenesis and dwarf (*CPD*) gene encodes C-3 oxidase, which is a rate-limiting enzyme that controls the synthesis of BRs.

**Methods:**

In this study, we used *StCPD* gene overexpression (T) and un-transgenic (NT) plants obtained from our former research to illustrate adaptive resistance to salt stress at levels of phenotype; cell ultrastructure, physiology, and biochemistry; hormone; and transcription.

**Results:**

Results showed the accumulation of 2,4-epibrassionolide (EBL) in T potatoes. We found that under high salt situations, the changed Na^+^/K^+^ transporter gene expression was linked with the prevalent ionic responses in T plants, which led to lower concentrations of K^+^ and higher concentrations of Na^+^ in leaves. Furthermore, RNA-sequencing (RNA-seq) data elucidated that gene expressions in NT and T plants were significantly changed with 200-mM NaCl treatment for 24 h and 48 h, compared with the 0-h treatment. Functional enrichment analysis suggested that most of the differentially expressed genes (DEGs) were related to the regulation of BR-related gene expression, pigment metabolism process, light and action, and plant hormone signal transduction.

**Discussion:**

These findings suggested that *StCPD* gene overexpression can alleviate the damage caused by salt stress and enhance the salt resistance of potato plantlets. Our study provides an essential reference for further research on BR regulation of plant molecular mechanisms in potatoes with stress tolerance.

## 1 Introduction

Salinity is among the most common environmental stresses that are involved in negatively influencing plant growth and development (Zhu, 2016); ion icing, osmosis, and oxidation; and DNA, RNA, and protein synthesis (van Zelm et al., 2020). There has been extensive research elucidating the influence of salinity on the phenotype, metabolism, and molecular levels of plants. For instance, the potato plants with *StCDPK2* (calcium-dependent protein kinases 2) overexpression mainly modulated osmotic and oxidative homeostasis and chlorophyll stability in the levels of transcription, photosynthesis, and metabolic profiling in response to salinity stimulation (Grossi et al., 2021). Moreover, when plants are exposed to salt stress, ion homeostasis and hormone levels are elevated (Yang and Guo, 2018). By decreasing Na^+^ concentrations and increasing K^+^ concentrations, plants keep a balanced cytosolic Na^+^/K^+^ ratio, protecting cells from damage under salt stress (Ketehouli et al., 2019). In the leaves of tissue culture seedlings of potatoes, the contents of ABA, BRs, and lignin increased markedly, whereas the contents of gibberellins (GAs) decreased remarkably under saline-alkali stress (Kang et al., 2021). Salt may lead to complex and severe injuries to plants. Conversely, plants have various mechanisms for adapting themselves to all external stimuli.

BRs play a substantial function in stimulating plant development by conquering the damaged effects of salinity (Rattan et al., 2022). BRs promote the processes of cell division and cell elongation to control plant growth (Nolan et al., 2020; Hu et al., 2016). Seif et al. (2014) reported that *Vitis vinifera* seedlings sprayed with 2,4-EBL increased photosynthesis, transpiration rate, pigments, and proline content to alleviate salinity stress. Researchers observed the pivotal role of BRs in keeping ion homeostasis by increasing the K^+^ concentrations and decreasing the Na^+^/K^+^ exposure to salt conditions (Sadeghi and Shekafandeh, 2014; Ahmad et al., 2018). BRs regulated stress response due to wide-ranging reactions such as changes in phenotype, concentrations of Na^+^/K^+^, key enzymatic reactions, protein synthesis, and diverse chemical defense compounds and nucleic acids (Baghel et al., 2019).

Furthermore, BR-mediated mechanisms for reducing the toxic effects of salinity also influence the transcription or translation of specific genes, activating the oxidative stress resistance ability of plant exposure to salt stress (Fariduddin et al., 2014; Cheng et al., 2015). The *CPD* gene encodes C-3 oxidase, and the *DWARF4* (*DWF4*) gene encodes a C-22 hydroxylase. They are cytochrome P450 enzymes and rate-limiting enzymes, which control the synthesis of BRs. In our former research, we found that *StDWF4* overexpression in potatoes could ameliorate the damage of salt stress and enhance salt resistance; however, interference expression of this gene in potato plants depressed salt tolerance mainly by destroying the antioxidative defense system and osmotic adjustment (Zhou et al., 2018).

In recent years, the critical roles of BRs in promoting tolerance to a complex sequence of abiotic stresses have been reported (Bajguz and Hayat, 2009; Divi et al., 2010; Bajguz and Piotrowska-Niczyporuk, 2014; Ahanger et al., 2018). In comparison, few studies have focused on the underlying mechanism of endogenous BRs in potato response to salt stress. Therefore, further studies are necessary to elucidate the response of endogenous BRs to the growth and development of salinity-tolerant potatoes.

In this study, we used the *StCPD* gene overexpression (T) and un-transgenic (NT) potato plants to explore the part of *StCPD* proteins of salt stress tolerance in potatoes. DEGs were identified and submitted to the GO database and KEGG databases for important information about the potential regulatory pathways in response to salt stress using RNA-seq. Furthermore, we found that overexpression of *StCPD* contributed to enhanced salt tolerance in the transgenic potatoes, indicating that endogenous BRs play a crucial part in the tolerance to salt stress. Therefore, integrated phenotype, physiology and biochemistry, hormone, and transcriptome analyses were performed to identify and characterize the pivotal regulatory genes, metabolites, and phytohormones involved in BR’s regulation of salt stress.

## 2 Materials and methods

### 2.1 Plant materials

One of the *StCPD* gene overexpression transgenic lines with stable expression levels named T and NT potato plants were obtained from our previous research; the relative expression of *StCPD* gene in T potato was 4.82 times higher than that of NT (Zhou et al., 2016). Seedlings were *in vitro* cultured in liquid Murashige–Skoog (MS) medium and under a 16 h day/8 h night cycle. (18°C) cycle, in environmental conditions at 25°C and 80% relative humidity (RH). Four-week-old seedlings were used for abiotic stress treatments. The plants were treated with 200 mmol·L^−1^ chloride (NaCl) at 0 h, 2 h, 6 h, 12 h, 24 h, and 48 h after treatments. All samples were taken at 6:00 pm. Three independent biological replicates were conducted for all samples under each treatment. Leaves of plants were immediately frozen at −80°C for subsequent ion concentration and EBL content determination and quantitative real-time PCR (qRT-PCR) tests. The phenotypic analysis (plant height, number of roots, maximum root length, stem thickness, and fresh weight) of NT and transgenic lines treated with NaCl at 0 h, 24 h, and 48 h was performed.

### 2.2 Observation of potato leaf ultrastructure

Leaf ultrastructure was observed by transmission electron microscopy following the protocols of Su et al. (2022). Briefly, fresh leaves of NT and T potatoes under salt stress for 0 h (CK) and 48 h were taken in the size of 2 mm × 2 mm, and the samples were quickly put into the centrifuge tubes containing electron microscope fixative for fixation, placed in the refrigerator at 4°C after 2 h at room temperature, and then washed with 0.1 M sodium phosphate buffer (PBS, pH 7.4) at 22°C thrice, for 15 min each. Then, it was fixed with 1% osmium tetroxide (w/v) at 4°C for 7 h and rinsed in 0.1 M PBS (pH 7.4) thrice, for 15 min each. Afterward, it was extracted in ethanol (30%, 1 h), 50% (1 h), 70% (1 h), 80% (1 h), 95% (1 h), and 100% (1 h, twice); ethanol: acetone (3:1, v/v) for 0.5 h, 1:1 for 0.5 h, and 1:3 for 0.5 h; and pure acetone for 1 h. Resin penetrated and embedded in acetone: EMbed 812 (epoxy resin, composed of methyl nadic anhydride (MNA), EPon-812, dodecenylsuccinic anhydride (DDSA), and 2,4,6-tris(dimethylaminomethyl)phenol (DMP-30), 3:1, v/v) for 2 h–4 h, 1:1 overnight, and 1:3 for 2 h–4 h at 37°C; pure EMbed 812 was embedded for 5 h–8 h at 37°C and poured into the embedding models, and then the tissues were inserted into the pure EMbed 812 and kept at 37°C overnight. Then, the tissues were polymerized at 65°C for 48 h. The resin blocks were cut to 60 nm–80 nm thickness on the ultra-microtome (EM UC6, Leica, Germany) and stained with 2% uranium acetate for 8 min, rinsed in 70% ethanol thrice, and then rinsed in ultrapure water thrice. In addition, it was stained with 2.6% lead citrate, and then the ultrastructure was observed by a transmission electron microscope (Hitachi 7800, Hitachi Ltd., Japan).

### 2.3 Measurement of MDA contents and enzymatic antioxidant activities

Methods of quantitative analysis of malondialdehyde (MDA) and antioxidant enzyme activities have been described in our previous article (Zhou et al., 2016).

### 2.4 Ion concentration determination

The Na^+^ and K^+^ contents of potato plants were determined as described by Xu et al. (2021). Leaves of transgenic and NT potatoes treated with salinity were oven-dried at 70°C for 3 days, and then they were ground into powder and incubated in a digested solution of 0.1 mol·L^−1^ acetic acid solution for 16 h. The powder was centrifuged at 12,000 rpm for 15 min. The supernatant was obtained. Na^+^ and K^+^ contents were measured by a flame photometer (F-500, Metash Shanghai, China).

### 2.5 Identification and quantification of EBL

The extraction of EBL was conducted according to Liu et al. (2022) with slight modification. 0.5-g leaves were ground into powder and soaked in 10 mL precooled methanol/formic acid (99/1, v/v), broken for 3 min at 40 Hz ultrasonication, left overnight at 4°C, centrifuged at 14,000 × *g* for 10 min, and then mixed with 1 mL supernatant and 9 mL UP H_2_O. The solution was purified on an SPE column activated with 3 mL methanol and 3 mL UP H_2_O. Then, 6 mL methanol/formic acid (99/1, v/v) was added for elution twice. The elution solution was collected. Finally, it was reconstituted in 1 mL methanol and filtered. 2-µL samples were quantified and identified by liquid chromatography tandem mass spectrometry (HPLC-MS, Agilent 1290, USA)/MS (QTRAP 6500, AB Sciex, USA). The EBL standard (≥98%, HPLC) was provided by Shanghai Yuanye Biotechnology Co., Ltd. (Shanghai, China).

### 2.6 Total RNA extraction and Illumina sequencing

The transcriptomics analysis of all potato plants treated at 0 h, 24 h, and 48 h was performed. RNA was isolated from the frozen leaf samples using TRIzol reagent (Tiangen Biotech, China) according to the manufacturer’s instructions. The quality and quantity were analyzed by NanoDrop® spectrophotometer 2000 (Thermo Fisher) and 1.0% denatured agarose gel electrophoresis. The mRNA was enriched with Oligo (dT) beads (Invitrogen, CA, USA). It was fragmented into short fragments. Then, mRNA template was reverse-transcribed into cDNA using random primers. The second strand of cDNA was generated. The cDNA fragments were purified using a QIAquick PCR extraction kit. Finally, the products were amplified by PCR and detected using agarose gel electrophoresis. RNA-seq was applied using a BGISEQ-500 sequencing platform from GeneRead Biotechnology Co., Ltd. (Wuhan, China). The transcriptomic raw data have been uploaded to the SRA database of NCBI (PRJNA995223).

### 2.7 Sequence filtration, assembly, and unigene expression analysis

Raw data were filtered to obtain clean reads, those with >10% unknown nucleotides, and >40% low-quality (Q-value ≤10) bases. Later, the clean reads were mapped to the reference transcriptome (Pham et al., 2020), and transcript abundance was examined using the method of Kallisto (Bray et al., 2016). Trinity was performed to assemble clean reads (Grabherr et al., 2011). The fragments per kilobase of exon per million mapped reads (FPKM) were employed to calculate and normalize gene expression levels (Mortazavi et al., 2008). In this study, levels of DEGs with a criterion of |FC| (|fold-change|) ≥1.5 and *P* ≤ 0.05 between transgenic and NT plants were used to identify DEGs using the DESeq2 package (https://bioconductor.org/packages/release/bioc/html/DESeq). Venn diagrams were created by Venny2.1 (https://bioinfogp.cnb.csic.es/tools/venny/index.html). Volcano plots were performed by Python seaborn (http://seaborn.pydata.org/), and the heatmap of DEGs was performed by TBtools (Chen et al., 2020).

### 2.8 Annotation of DEGs and analysis of gene cluster

Unigenes were annotated using the databases of NCBI non-redundant Protein (NR), Universal Protein (UniProt), Swiss-Prot protein, euKaryotic orthologous groups of proteins (KOG), Kyoto Encyclopedia of Genes and Genomes (KEGG), and gene ontology (GO) by a BLASTx procedure with an e-value ≤10^−5^ (Conesa et al., 2005), respectively. Molecular Evolutionary Genetics Analysis (MEGA) 7.0 was employed to analyze the gene cluster.

### 2.9 Conformation of transcriptome data by qRT-PCR

To verify the transcriptome data, the *ef1a* gene (GenBank accession no. AB061263) was selected as the endogenous reference gene. There were 17 upregulated DEGs (UR) and six downregulated DEGs (DR) selected for qRT-PCR verification. Total RNA was extracted using the RNA Easy Fast Plant Tissue Kit (TIANGEN, DP452) following the manufacturer’s instructions. The integrity of total RNA was analyzed by 1.0% agarose gel electrophoresis. The information about primer sequences of selected DEGs (**Supplementary Table 1**) was designed using NCBI (https://www.ncbi.nlm.nih.gov/tools/primer-blast/) and synthesized by Sangon Biotech (Shanghai, China). First-strand cDNA was synthesized using the FastKing cDNA Synthesis Kit (TIANGEN, KR118-03) following the manufacturer’s instructions. The qRT-PCR gene expression was inspected using a SuperReal PreMix Plus (SYBR Green) (TIANGEN, FP205-03). Reactions were carried out on a LightCycler (LightCycler96 Real-Time PCR, Roche, Switzerland) by the default cycling conditions (15 min at 95°C and 40 cycles of 10 s at 95°C, 20 s at 60°C, 30 s at 72°C and 95°C for 15 s, 60°C for 1 min). The melting curve analysis was used to examine the specificity of each amplification, and the relative expression levels were calculated by the 2^−ΔΔCt^ quantitative method (Ct, cycle threshold value of target gene) (Willems et al., 2008).

## 3 Statistical analysis

All measurements were conducted using three biological replicates. One-way ANOVA and Duncan’s multiple comparison tests were used to analyze statistically. The software SPSS version 22.0 (SPSS, USA) was used, with *P* ≤ 0.05 considered for all experiments at a significant level.

## 4 Results

### 4.1 Phenotypic parameters analysis of the potato plants under salt stresses

**Figure 1:**
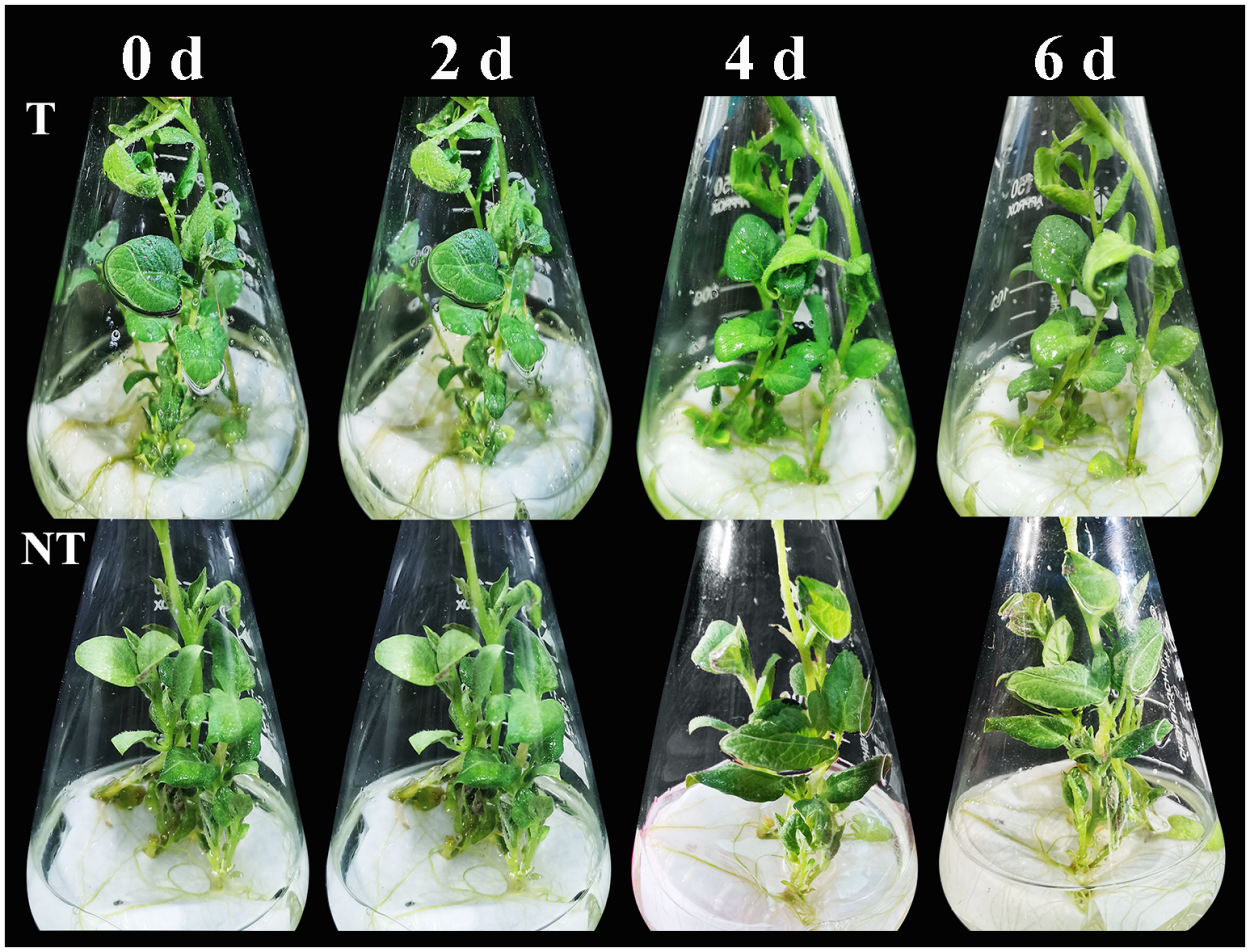
Phenotype changes in NT and T potato plantlets cultured under salt stresses.

After 2-day salt stress, transgenic and NT potato plantlets grew well, and there was no notable phenotypic difference. Four days later, we surveyed slight wilting of the NT plants, and the leaf edge became yellow. After 6 days of salt stress, the shoots and leaves of the NT plants severely withered, and the leaf edge became yellow, whereas the transgenic shoots and leaves were only mildly withered (**Figure 1**). The plant height, stem thickness, root length, fresh weight and size, and fresh weight of T potatoes were significantly higher than those of NT plants under salt stress (**Tables 1**, **2**). These phenotypic variations indicated that *StCPD* gene overexpression potatoes were resistant to salt stress.

**Table 1:** Effects of salt stress on fresh and dry weight between NT and T potato leaves.

Treatment time (h)	Leaf fresh weight (g/plant)		Leaf dry weight (g/plant)	
	NT	T	NT	T
0	0.060 ± 0.009^b^	0.090 ± 0.032^a^	0.693 ± 0.098^c^	0.997 ± 0.049^b^
24	0.061 ± 0.004^b^	0.111 ± 0.028^a^	0.795 ± 0.118^c^	1.195 ± 0.020^a^
48	0.057 ± 0.003^b^	0.101 ± 0.012^a^	0.707 ± 0.112^c^	1.153 ± 0.117^a^

The method of significant difference analysis is the same as in **Figure 3**. Bars = standard errors (n = 9, i.e., 3 biological repeats × 3 technical repeats). Different letters indicate significant differences between different treatments (P<0.05), same as below.

**Table 2:** Effects of salt stress on plant height, root length, and stem diameter of NT and T potato leaves.

Treatment time (h)	Plant height (cm)		Root length (cm)		Stem diameter (mm)	
	NT	T	NT	T	NT	T
0	8.80 ± 0.212^b^	9.92 ± 0.522^a^	9.08 ± 0.327^d^	13.64 ± 0.365^b^	1.43 ± 0.031^b^	1.55 ± 0.059^ab^
24	8.22 ± 1.057^bc^	9.96 ± 0.241^a^	9.84 ± 0.329^c^	13.78 ± 0.572^b^	1.20 ± 0.061^c^	1.58 ± 0.030^ab^
48	7.86 ± 0.305^b^	9.62 ± 0.217^a^	9.78 ± 0.460^c^	14.24 ± 0.439^a^	1.16 ± 0.035^c^	1.67 ± 0.249^a^

The method of significant difference analysis is the same as in **Figure 3**. Bars = standard errors (n = 9, i.e., 3 biological repeats × 3 technical repeats). Different lowercase letters indicate significant differences between different treatments (P<0.05).

### 4.2 Cell ultrastructure changes of NT and T potatoes under salt stresses

**Figure 2:**
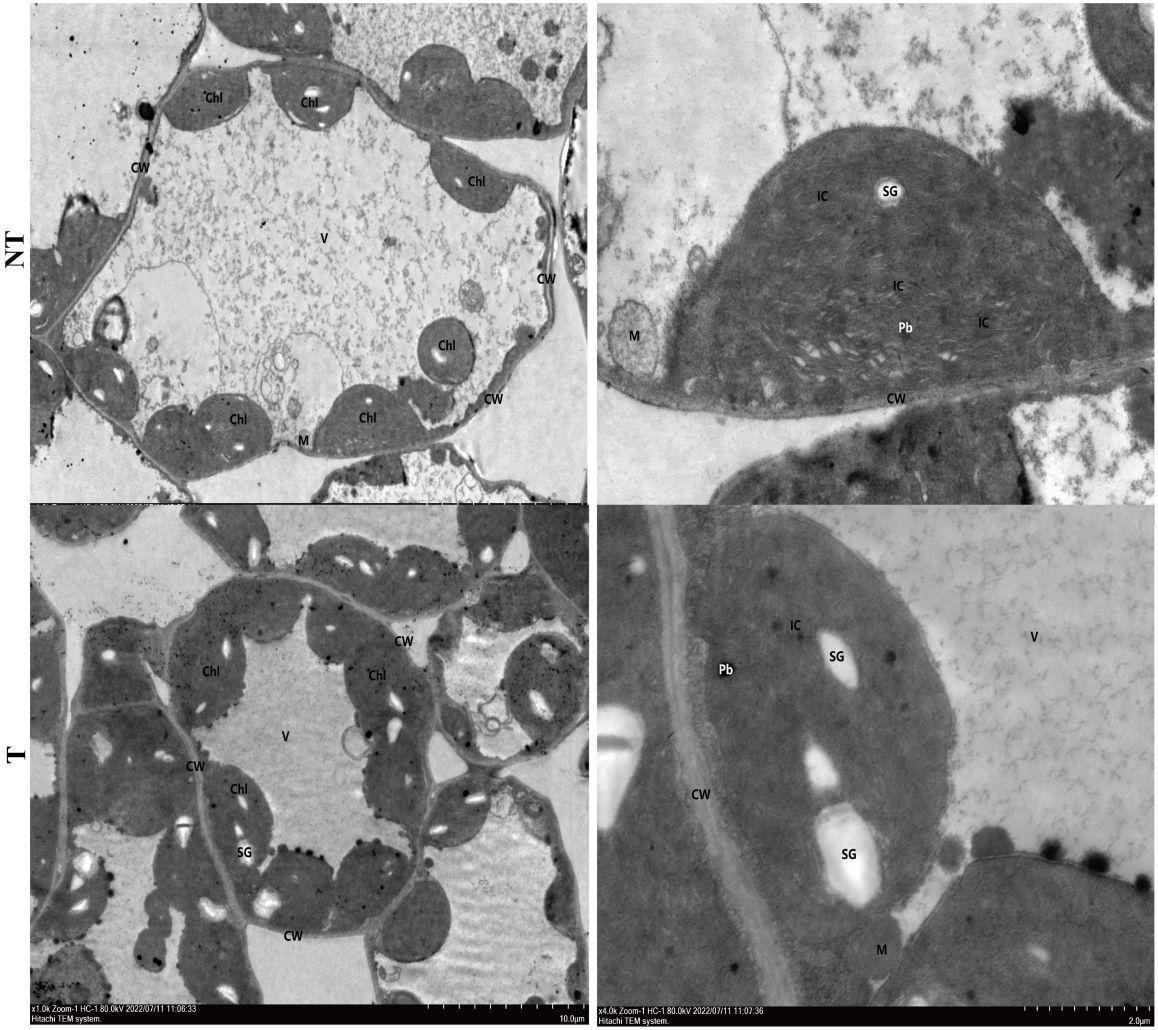
Cell ultrastructure of NT **(A, B)** and T **(C, D)** potato leaves under 48-h salt treatment.

The cell ultrastructure of potato leaves treated with salt stress for 48 h is shown in **Figure 2**. The damage of NT potato leaves was relatively obvious compared with that of the control (0 h). Organelles were obviously free. Chloroplasts (Ch) were abundant, but the membrane structures were loose, and the lamellar structure of the basal granule-like cysts (IC) was disordered and loose. A small number of mitochondria (M) obviously began to be swollen. The matrix was dissolved, and the cristae disappeared. Large areas of vesicle (V) membranes were broken and disintegrating, and a large number of flocculants and free organelles could be seen. The cell ultrastructure of T potato leaves was relatively normal compared with that of the control, with abundant cytoplasmic organelles and normal structure. Chloroplasts (Ch) were abundant, with intact membrane structures, and basaloid cyst (IC) lamellae structures were clear. Mitochondria (M) membranes were intact and cristae were present, and vesicles (V) had intact membranes (**Figure 2**).

### 4.3 Physiological response of NT and T potatoes under salt stresses

**Figure 3:**
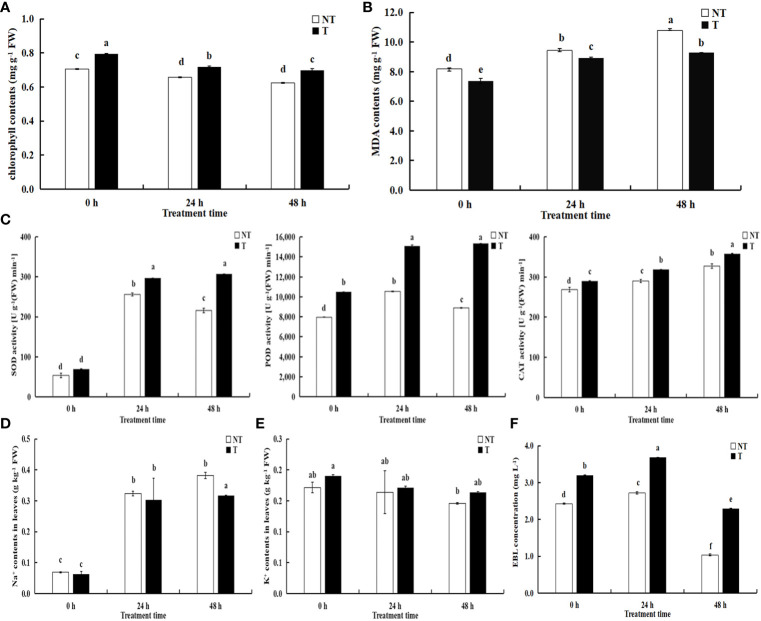
Effects of salt stress on physiological indicators **(A)** chlorophyll contents; **(B)** MDA contents; **(C)** SOD, POD, CAT activities), Na^+^ **(D)**, K^+^ **(E)**, and EBL concentrations **(F)** in NT and T potato leaves. Significant differences among means over the period of salt-stress treatments were determined according to Duncan’s test at *P* < 0.05 (different lowercase letters). Bars = standard errors (n = 9, i.e., 3 biological repeats × 3 technical repeats).

All the parameters related to chlorophyll fluorescence significantly declined under salt treatment. The chlorophyll in transgenic leaves was strikingly more than that in NT under salt conditions. After 24 h and 48 h of salt treatment, the growth of transgenic potato plants was better, but the chlorophyll contents decreased by 8.60% and 10.54%, compared with NT under salt stress (**Figure 3A**). Under salt stress, the activities of peroxidase (POD), superoxide dismutase (SOD), and catalase (CAT) in the T leaves were higher than NT potatoes and the activities of POD and SOD in NT increased first then decreased with extension of the salt-treated time, whereas the activities in T potato continued to increase (**Figure 3C**). The CAT activities and MDA contents in NT and T leaves increased, but the MDA contents in T leaves was lower than those in NT potatoes (**Figure 3B**).

### 4.4 Analysis of ion concentrations

The Na^+^ contents in the leaves of transgenic plants decreased by 6.44% and 17.09%, respectively compared with NT under 200 mM NaCl treatment of 24 h and 48 h, and Na^+^ concentrations in transgenic plants were slightly lower than NT without treatment (*P* < 0.05). However, K^+^ concentrations in the leaves of transgenic plants increased by 4.58% and 11.98%, respectively, compared with NT under salt treatment of 24 h and 48 h. Moreover, Na^+^ and K^+^ concentrations in both transgenic and NT plants enhanced with the aggravated degree of stress (**Figures 3D, E**).

### 4.5 Changes in EBL levels

We compared variations in EBL contents between transgenic and NT plants responding to salt stress using UPLC-MS/MS. We found significant differences in the former compared with the latter, and the EBL contents in T leaves were always higher than those in NT. With the extension of stress time, the EBL levels increased followed by a decrease in both transgenic and NT plants with salt stress time extension (**Figure 3F**). Therefore, we speculated that BR-related genes participate in the regulation of the synthesis and signal transduction of BRs.

### 4.6 Analysis of transcriptome sequencing and global gene

To illuminate molecular mechanisms of BR regulation in potatoes, a comparison of gene transcription in transgenic and NT plants at different salt stress was performed. A total of around 6 GB of clean data was generated through RNA-seq. After data filtering, robust data were obtained and 40.07–53.33 million clean reads were generated for the samples. In contrast, the Q30 in all samples was more remarkable than 92.37%, the GC content of each sample was 42.08%–44.66%, and over 78.60%–80.03% of clean reads were successfully aligned to the potato DMv6.1 reference genome. A total of 32,917 unigenes were obtained (**Supplementary Tables 2, 3**).

**Figure 4:**
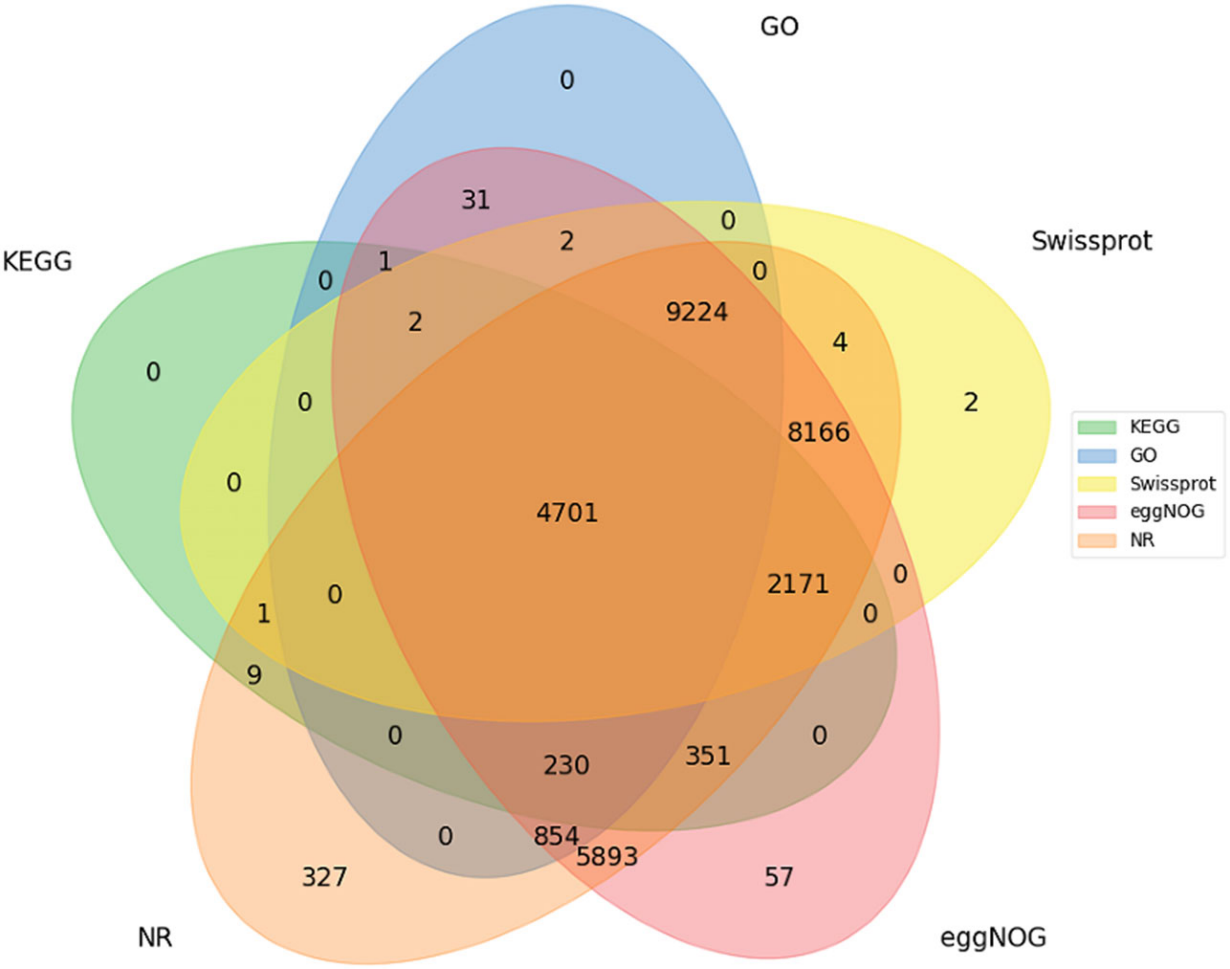
Functional annotations for all unigenes.

**Figure 5:**
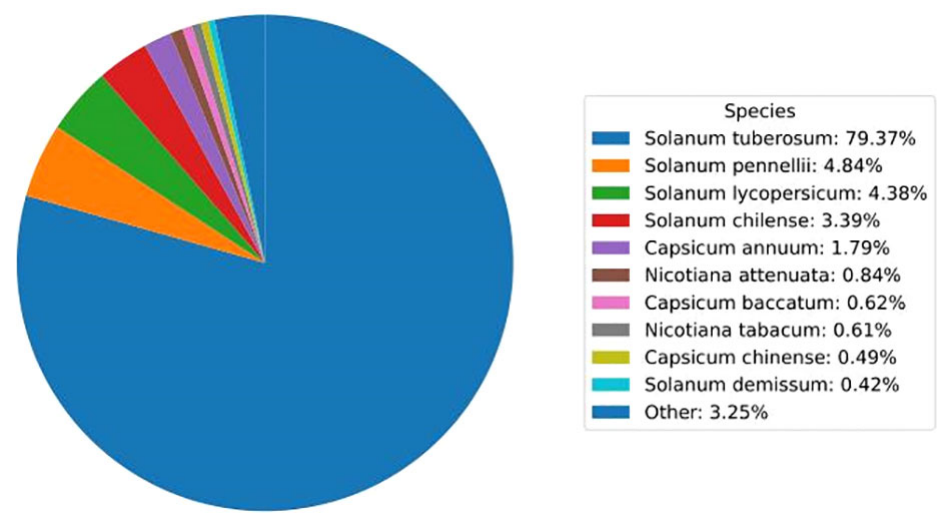
Top 10 plant species distribution of unigenes against the NR database.

**Figure 6:**
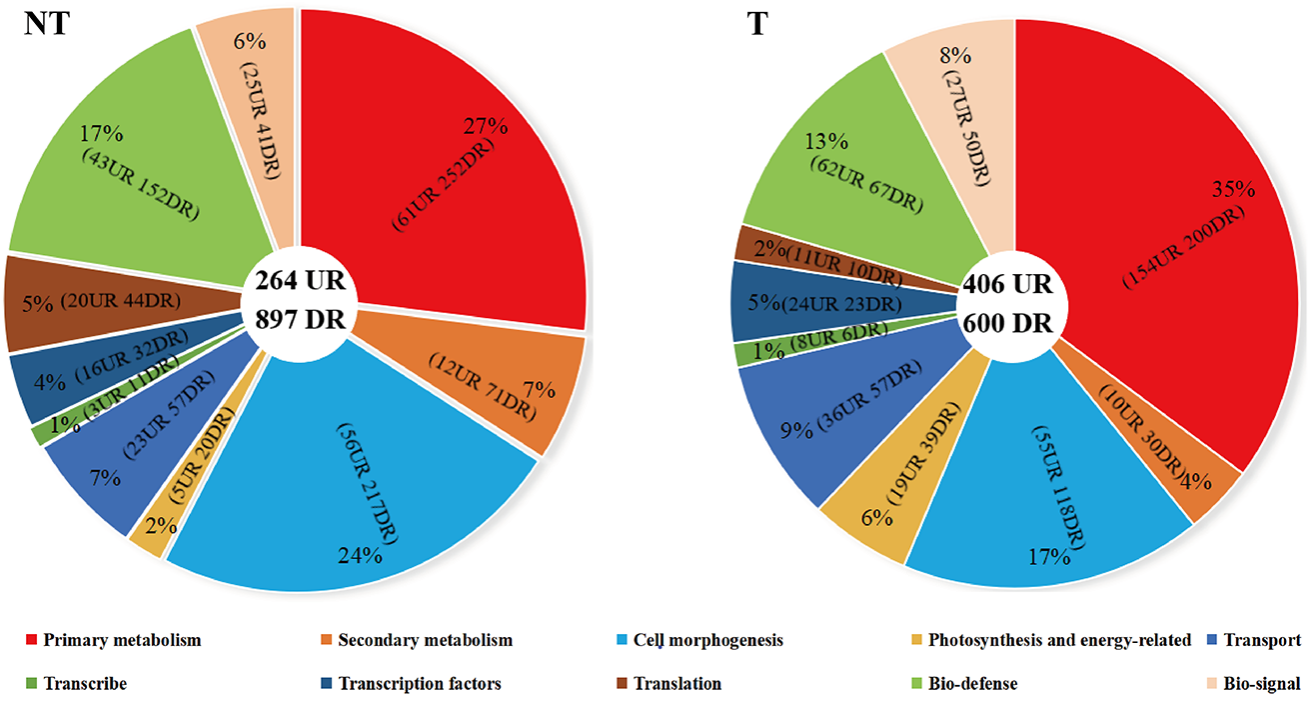
Distribution and classification of DEGs in NT and T potatoes.

Unigenes were annotated against the databases, including NR, SwissProt, UniProt, KEGG, KOG, and GO (**Figure 4**, **Supplementary Table 3**), and they were distributed in the top 10 plant species: *Solanum tuberosum*, *Solanum pennellii*, *Solanum lycopersicum*, *Solanum chilense*, *Capsicum annuum*, *Nicotiana attenuata*, *Capsicum baccatum*, *Nicotiana tabacum*, *Capsicum chinense*, and *Solanum demissum* (**Figure 5**). A total of 1,669 and 1,362 DEGs were generated in NT and T potatoes under salt stress, 466 and 294 genes were unidentified, and 1,203 and 1,068 identified DEGs, of which 1,161 (264 UR, 897 DR) and 1,006 (406 UR, 600 DR) DEGs have biological properties, respectively (**Figure 6**).

### 4.7 GO analysis of DEGs in transgenic and NT potato plants

The GO annotation results showed that DEGs were present in three major categories: biological processes, cellular components, and molecular functions, as shown in **Supplementary Figure 1**. It demonstrated the top 30 entries of GO enrichment abundance, with the same entries of differential gene enrichment in transgenic and NT potatoes but different numbers of DEGs. The number of DEGs was mainly distributed on metabolic pathways including photosynthesis, light reaction, generation of precursor metabolites and energy, and chlorophyll binding which varied widely (**Supplementary Figure 1**).

**Figure 7:**
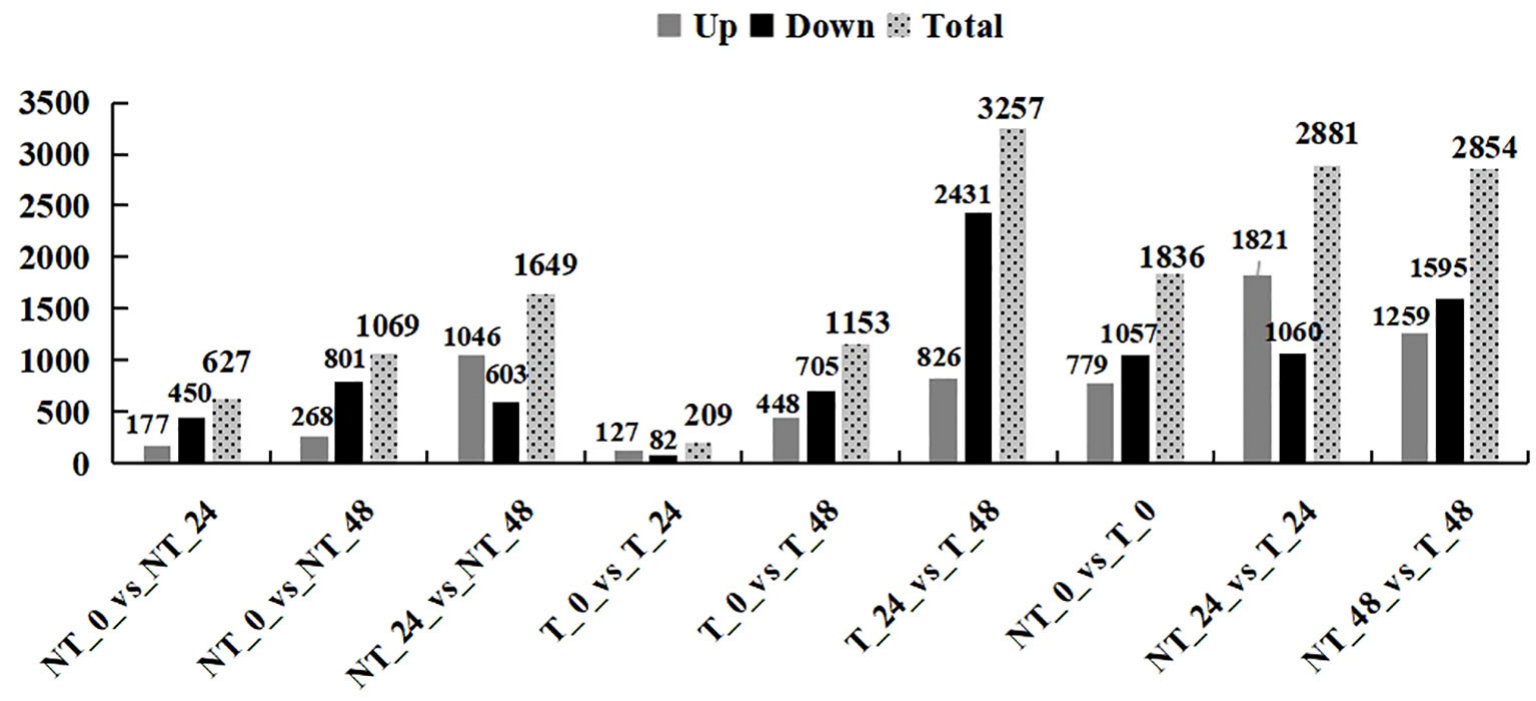
Statistics of the number of differential genes.

In total, 1,836 (NT_0_vs_T_0; 779 upregulated and 1,057 downregulated), 2,881 (NT_24_vs_T_24; 1,821 upregulated and 1,060 downregulated), and 2,854 (NT_48_vs_T_48; 1,259 upregulated and 1,595 downregulated) genes were differentially expressed in transgenic plants compared with the NT groups in 0 h, 24 h, and 48 h salt treatment, respectively (**Figure 7**).

**Figure 8:**
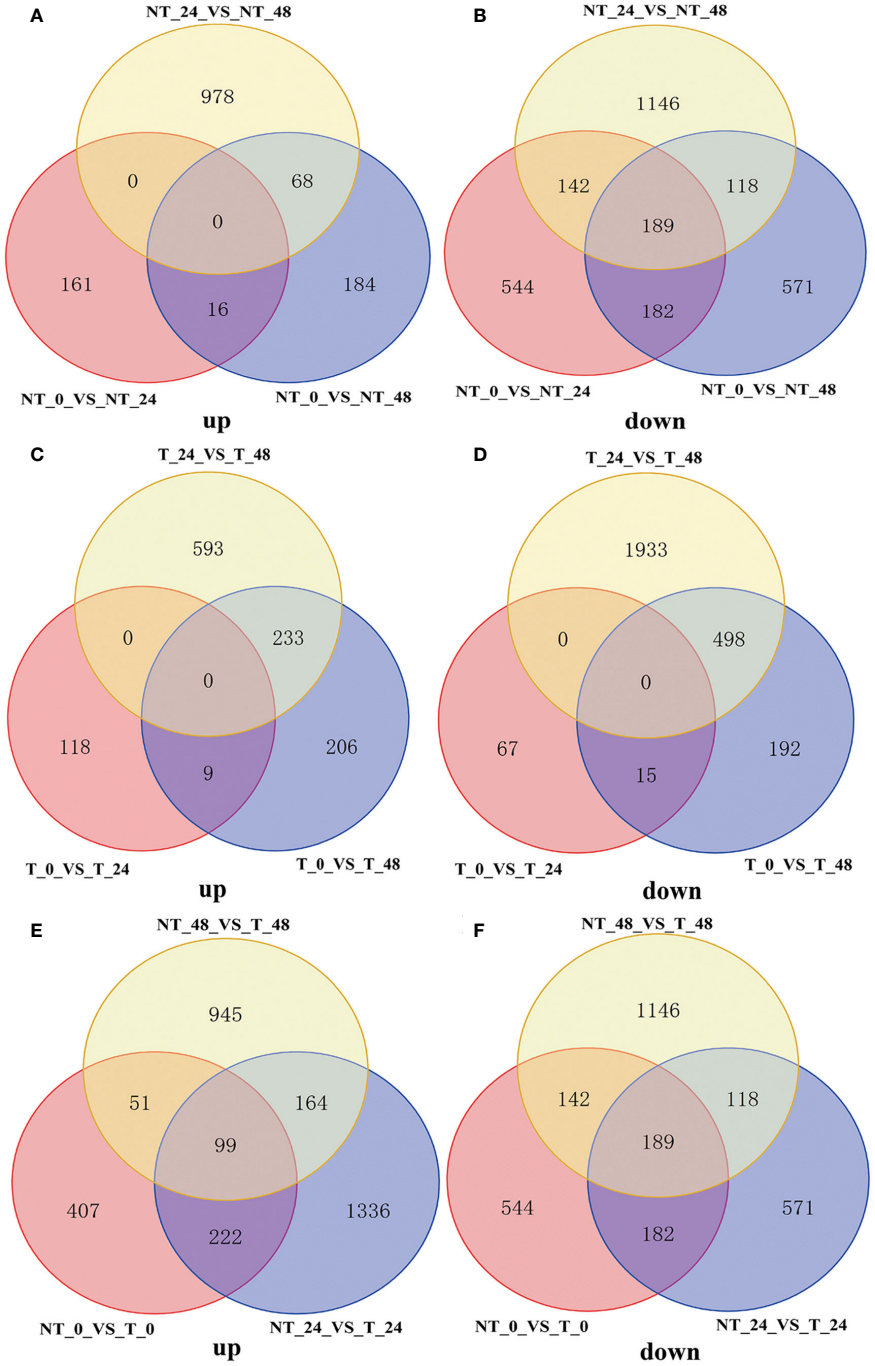
Venn diagram of DEGs under different comparison groups. **(A)** Upregulated DEGs of NT_0_vs_NT_24, NT_0_vs_NT_48, and NT_24_vs_NT_48; **(B)** downregulated DEGs of NT_0_vs_NT_24, NT_0_vs_NT_48, and NT_24_vs_NT_48; **(C)** upregulated DEGs of T_0_vs_T_24, T_0_vs_T_48, and T_24_vs_T_48; **(D)** downregulated DEGs of T_0_vs_T_24, T_0_vs_T_48, and T_24_vs_T_48; **(E)** upregulated DEGs of NT_0_vs_T_0, NT_24_vs_T_24, and NT_48_vs_T_48; **(F)** downregulated DEGs of NT_0_vs_T_0, NT_24_vs_T_24 and NT_48_vs_T_48.

As shown in **Figures 8A, B**, NT potatoes at 24 h and 48 h of salt stresses shared 16 upregulated genes and 182 downregulated genes, and T potatoes at 24 h and 48 h of salt stresses shared 9 upregulated genes and 15 downregulated genes (**Figures 8C, D**). In the NT_0_vs_T_0, NT_24_vs_T_24, and NT_48_vs_T_48, 99 shared upregulated genes and 189 shared downregulated genes were found (**Figures 8E, F**).

**Figure 9:**
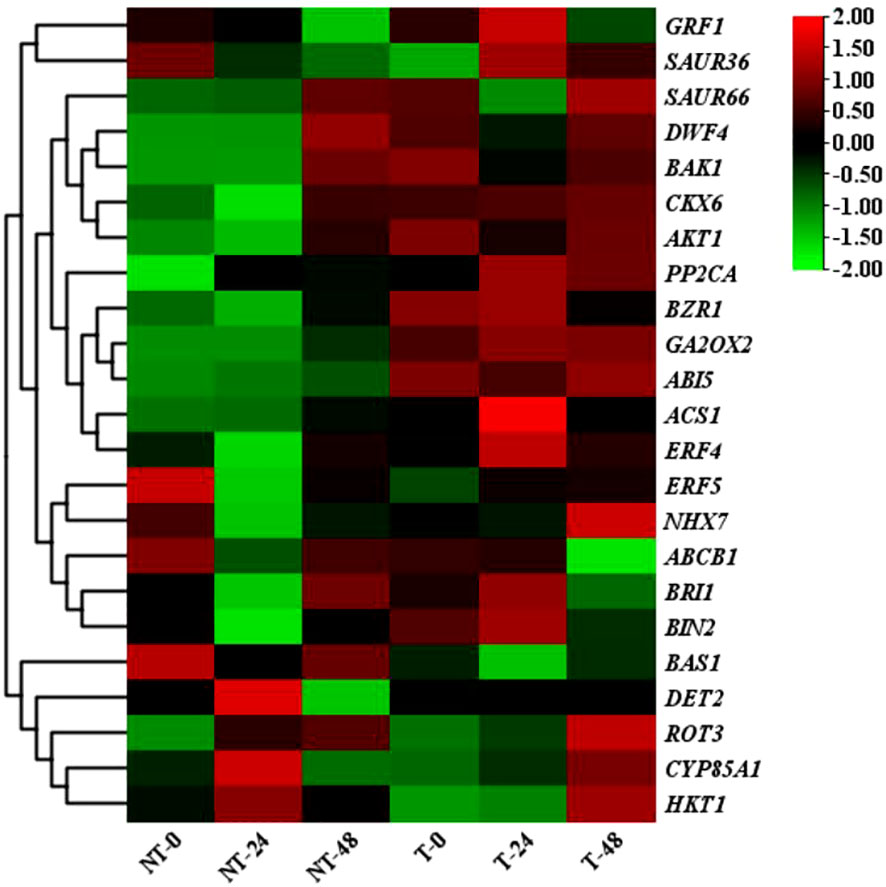
Heat map of differential gene expression in NT and T potatoes, based on FPKM values.

The clustering heatmap showed considerable differences in gene expression levels between transgenic and NT plants (**Figure 9**). These genes were mainly involved in the regulation of plant hormone signal transduction gene, phenylpropanoid biosynthesis gene, and photosynthesis gene expression. Through KEGG enrichment analysis, the pathways significantly enriched in transgenic plants including light and action, plant hormone signal transduction, photosynthesis-antenna protein, and MAPK signaling pathway (**Supplementary Figures 1**-**3**).

### 4.8 TFs involved in BR biosynthesis as well as salt tolerance

**Figure 10:**
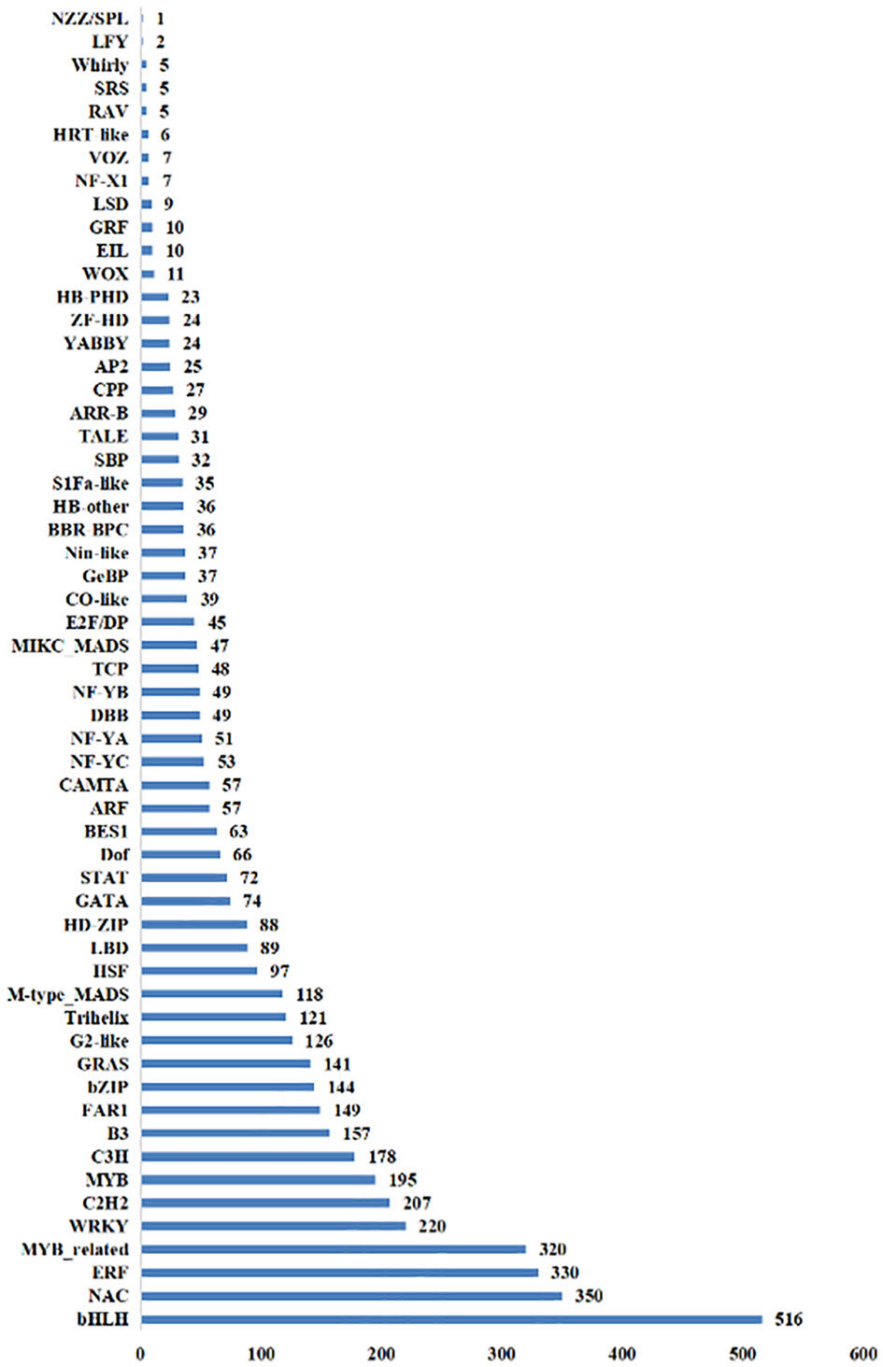
The total number of transcription factors screened out of differential genes in NT and T potato leaves under NaCl stress.

There were 52 TFs with a total of 1,074 DEGs identified in the NT_0_vs_T_0 group, and bHLH, ERF, and NAC gene families were the most abundant with 111, 101, and 87 DEGs, respectively. In the NT_24_vs_T_24 comparison group, 55 TFs with a total of 1,566 DEGs were identified, including bHLH, NAC, and MYB_related gene families with 148, 109, and 107 DEGs, respectively. There were 55 TFs with 1,542 DEGs identified in the NT_48_vs_T_48 comparison group. The most abundant families were bHLH, NAC, and ERF, with 168, 129, and 112 DEGs, respectively (**Figure 10**).

### 4.9 DEGs of potato plants responded to salt stresses

With the extension of the salt-treated time, the number of DEGs gradually increased in T and NT potatoes. A total of 3,345 DEGs were identified, of which 44.6% were upregulated and 55.4% were downregulated. Notably, under 200 mM NaCl, the DEGs in transgenic and NT potatoes were lower by 209 and 627 at 24 h and higher by 1,153 and 1,069 at 48 h salt treatment, respectively (**Figure 7**).

KEGG enrichment analysis was performed on DEGs of both NT and T plants with top 20 pathways. Two pathways of ubiquitin-mediated proteolysis and aminoacyl-tRNA biosynthesis were significantly enriched in NT at 24 h of salt stress. However, the highest enrichment was in photosynthesis-antenna protein and plant hormone signal pathways of transgenic plants. At 48 h of salt stress, the most enriched pathways in NT and T were plant hormone signal transduction, and starch and sucrose metabolism (**Supplementary Figures 2**, **3**).

### 4.10 DEGs linked with BR biosynthesis, signal transduction, and metabolism pathway

**Figure 11:**
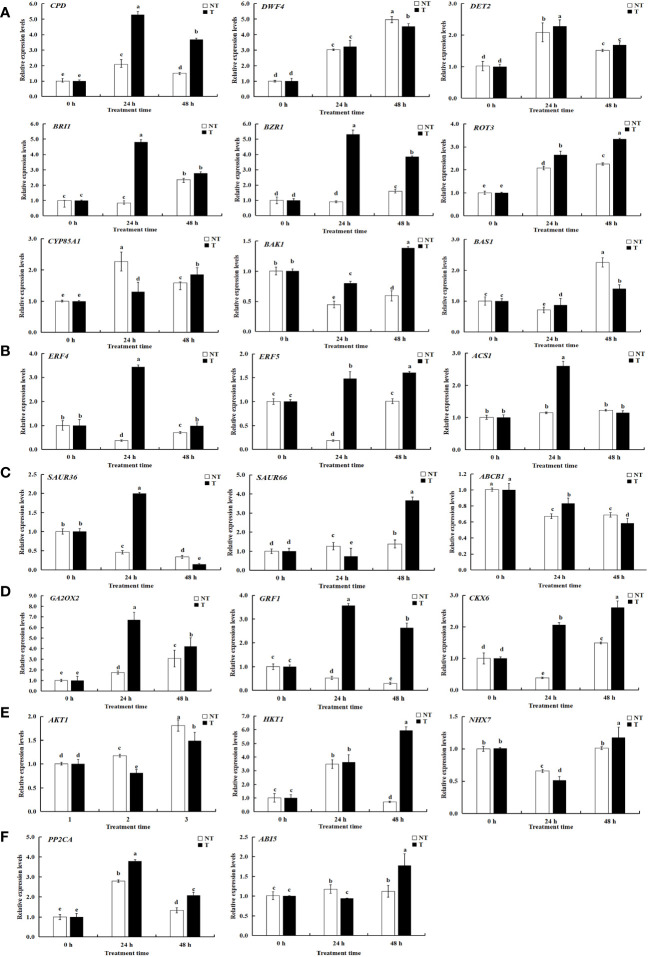
Relative expression levels of selected genes validated by qRT-PCR. A-F represents differentially expressed genes related to BRs, ETH, IAA, ABA, GA-CTK, and osmoregulation. Significant differences among means over the period of salt-stress treatments were determined according to Duncan’s test at P < 0.05 (different lowercase letters). Bars = standard errors (n = 9, i.e., 3 biological repeats × 3 technical repeats).

KEGG pathway analysis was employed to distinguish the complex biological functions corresponding to DEGs. Four DEGs directly participated in upstream or downstream of the BR biosynthesis pathway, including *DWF4*, *DET2*, *ROT3*, and *CYP85A1*. RELs of these genes have upregulation of 4.54-, 1.99-, and 1.97-fold for *DWF4*, *DET2*, and *ROT3*, with downregulation of 0.40-fold for *CYP85A1*, in transgenic compared with NT plants at 0-h treatment time. Three DEGs were directly involved in BRs’ signal transduction, namely, *BZR1*, *BRI1*, and *BAK1*. The RELs of these genes were upregulated by 2.35-, 2.46-, and 1.36-fold for *BZR1*, *BRI1*, and *BAK1*, respectively, in transgenic compared with NT plants at 0-h treatment time. The *BAS1* gene participated in BR metabolism; the RELs were 0.68-fold in transgenic vs. NT at 0-h treatment time (**Figures 9**, **11A**; **Supplementary Table 4**). These results exhibited these pathways; particularly, BR biosynthesis may be critically important in potato salt stress resistance.

### 4.11 DEGs linked with Na^+^/K^+^ transporter gene expression

To determine the related genes of Na^+^ and K^+^ in the roots of transgenic and NT potatoes under different salt conditions, the genes related to Na^+^/K^+^ transporter were identified from the data of RNA-seq, including one high-affinity K^+^ transporters (*HKT1*), one vacuole membrane Na^+^/H^+^ reverse transporter (*NHX7*), and one potassium channel protein (*AKT1*) (**Figure 9**, **Figure 11E** and **Supplementary Table 4**).

Specifically, *HKT1* was higher in transgenic plants at 48 h treatment time, but not in NT. However, differences in *HKT1* expression between transgenic and NT potatoes were insignificant at 0-h and 24-h stress treatments. Notably, *NHX7* was upregulated by 310.53- and 551.69-fold in transgenic plants at 24 h and 48 h. Moreover, the *AKT1* in transgenic plants was upregulated under all salt stress compared with NT.

### 4.12 DEGs linked with photosynthesis and energy-related gene expression

**Figure 12:**
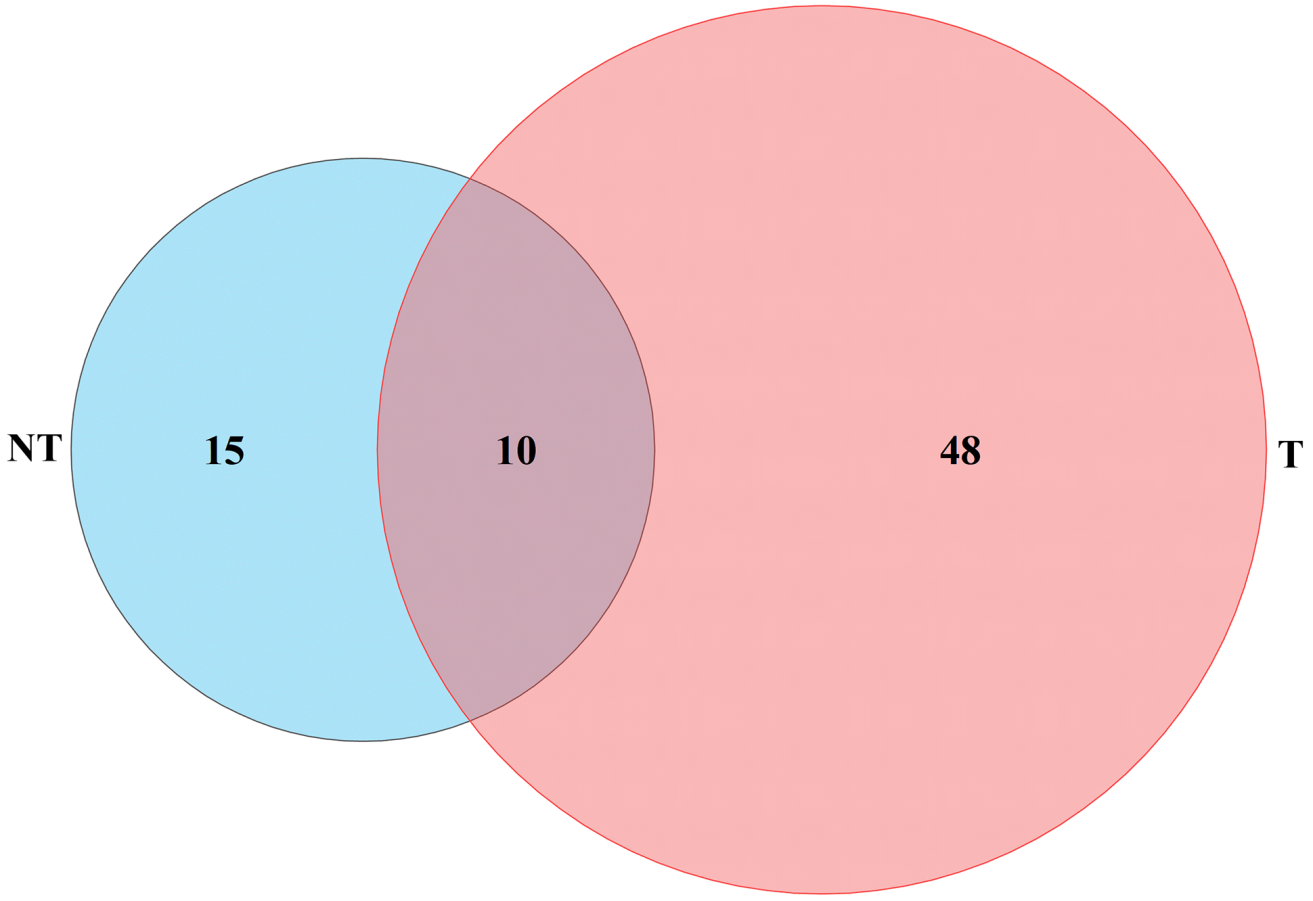
Photosynthesis and energy-related genes differentially expressed under salt stresses.

Studies have shown that genes in photosynthesis took a pivotal part in plant salt stress (Rattan et al., 2014). We found that 25 and 58 DEGs were associated with photosynthesis in NT and transgenic plants under salt stress; of these, 10 genes functioned in both NT and T plants. Five genes were downregulated and five genes were upregulated in NT, and seven genes were upregulated and three genes were downregulated in T potatoes at 24 h of salt stress. Two genes were upregulated and eight genes were downregulated in NT, and one gene was upregulated and nine genes were downregulated in transgenic potatoes at 48 h of salt stress (**Figure 12**), indicating that salt stress can induce photosynthetic response genes, to ensure the normal operation of photosynthesis and improve salt tolerance.

### 4.13 Quantitative real-time validation of DEGs

A total of 23 DEGs were verified by qRT-PCR. Of these, 9 DEGs were linked with BRs biosynthesis and the other 14 DEGs were correlated with salt stress resistance from both the transgenic and NT plants, using qRT-PCR to verify transcriptome data. The expression patterns of most of the selected genes were similar to those observed by RNA-seq, demonstrating the reliability of transcriptome data (**Figures 9**, **11**; **Supplementary Table 5**).

## 5 Discussion

BRs are vital in plants’ ability to sense salt stress (Dong et al., 2020). We previously demonstrated that physiological and biochemical variations in *StDWF4*, which was also critical for BR biosynthesis and the contents of endogenous BRs, as well as its enhanced or inhibited expression in plants, provoked salt responses in potatoes (Zhou et al., 2018). It is well-known that BRs regulate plant growth and development to alleviate the negative impact of salinity in plants (Khalid and Aftab, 2016; Fahim et al., 2020). However, brassinolide-induced stress resistance is a rather complex process involving dynamic changes in various intrinsic factors (Sharma et al., 2013). The molecular mechanisms of any possible interactions between BRs and salt tolerance remain unknown.

Emerging evidence supports the immense significance of exogenous BRs on the phenotype, physiological and biochemical parameters, and molecular mechanisms of plants under various abiotic stresses such as drought and salinity (Yusuf et al., 2016; Rattan et al., 2022). Therefore, in this study, we focused on the contents of endogenous BRs, Na^+^ and K^+^ concentrations, and levels of the transcription of potato plantlets before and after salt stress to elucidate the molecular basis of salt tolerance in transgenic and NT potatoes. These results provide further insight into the regulating mechanism of BRs in potato salt stress.

### 5.1 Physiological parameter differences in salt tolerance between transgenic and NT plants

Researchers have stated that reduced photosynthetic pigments, due to increased chlorophyll activities, caused the destabilization of chlorophyll protein complex-associated pigments under salt stress (Rasool et al., 2013). Chlorophyll contents differed with different treatment time points between the transgenic and NT plants. Overall, the reduction in total chlorophyll in NT lines was higher than in transgenic lines caused by salt stress, demonstrating that *StCPD* protected photosynthetic pigments and avoided chlorophyll degradation. The same results were also reported in *Arabidopsi*s and Poplar (Bancos et al., 2002; Feng et al., 2021b). The application of BRs to overcome adverse impacts of salinity on the growth of *Zea mays* was associated with enhanced photosynthesis activity (Rattan et al., 2014). Supplementation of plants with EBL under salt stress-induced enhances chlorophyll synthesis, thereby increasing their photosynthetic potential, alleviating damage and promoting the plant tolerance to salt (Sun et al., 2015). Seedling-sprayed 2,4-EBL (10^−6^ M) helped salt-treated watermelon to maintain photosynthetic enzymes, including rubisco, influencing rubisco carboxylation rates and RuBP regeneration, to enhance photosynthesis under salt stress (Cheng et al., 2015).

When concentrations of Na^+^ and Cl^−^ in the growing environment were more than a certain range, plants would undergo varying degrees of salt stress (Ismail et al., 2014; Qin and Huang, 2020). Furthermore, high concentrations of Na^+^ in plants inhibited the absorption of K^+^, resulting in ion imbalance, thereby inducting osmotic and oxidative stress (Alam et al., 2018). The maintenance of cellular Na^+^/K^+^ homeostasis was important to determine the survival ability during salt stress in plants (Yang and Guo, 2018). To accommodate salt stress, plants have established well self-regulated mechanisms to keep ionic homeostasis (van Zelm et al., 2020). Na^+^ and MDA contents in leaves can mirror the ability of a plant’s tolerance to salt stress (Tong et al., 2022). As shown in **Figures 4A, B**, Na^+^ contents in NT plants were higher than those in the *StCPD* transgenic potato lines at 48 h salt stress, and MDA in the former was higher than those in the latter in all of the treatments. The application of bioactive EBL could effectively promote plant salinity tolerance in barley seedlings by reducing K^+^ efflux *via* depolarization-activated K^+^ channels (Azhar et al., 2017). Sadeghi and Shekafandeh (2014) evaluated loquat seedlings treated with different degrees of 2,4-EBL under salt stress and found a dramatic effect of foliar-applied 2,4-EBL on contents of Na^+^, K^+^, and Cl^−^ in leaves. Alam et al. (2018) observed that salinity promoted the Na^+^ uptake in root and shoot coupled with a decline in the uptake of K^+^. However, the external application of EBL decreased Na^+^ accumulation and enhanced the uptake of K^+^. Similarly, the study supported the significant part of BRs in mediating salinity tolerance in tomatoes of the ion homeostasis by increasing the K^+^ concentrations and reducing the Na^+^/K^+^ ratio under salt conditions (Ahmad et al., 2018).

Gupta et al. (2017) showed that the application of exogenous EBL reduced Na^+^ accumulation by approximately 44% and raised K^+^ contents by approximately 122% in *Brassica juncea* roots. Hu et al. (2016) found that brassinolide (BL) application alleviated salt stress by maintaining Na^+^/K^+^ homeostasis in potatoes. Therefore, we speculated that *StCPD* overexpression enhanced the contents of EBL, thus positively regulating salt tolerance in the transgenic potatoes, which is consistent with previous investigations (Feng et al., 2021a). The above results suggest that the regulation of Na^+^/K^+^ balance under salt stress may provide an effective approach for regulating salinity tolerance in potatoes.

In addition, the activity of the SOD enzyme continually enhanced in transgenic potatoes but increased first and then in NT plants. These results indicated that the NT potatoes were injured by salinity more severely than transgenic plants.

### 5.2 EBL contents changed between transgenic and NT plants under salt stresses

BRs played a promising role in various aspects of plant development (Gudesblat and Russinova, 2011; Sreeramulu et al., 2013; Baghel et al., 2019). Especially, increasing evidence indicated that the brassinosteroid analog, the EBL, is involved in salt stress response in numerous plant species (Derevyanchuk et al., 2015; Khalid and Aftab, 2016; Zheng et al., 2016). Exogenous application of EBL caused variation of protein, proline, and MDA contents and antioxidant enzyme activities, as well as enhanced expression of encoded genes in BR synthesis and salt-responsive genes, and enhanced salt tolerance in rice (Sharma et al., 2013). In this study, we further explored the effects of *StCPD* overexpression on the salt stress of potatoes, resulting from the variation of endogenous EBL levels.

The BR contents of the transgenic plants were changed significantly compared with wild-type plants (**Figure 5**). Thus, the biosynthesis and metabolism of the BRs were varied and adjusted in the transgenic lines. Overexpression of the key gene *PtoDWF4*, which was also responsible for the biosynthesis of BRs, caused the contents of BRs insignificantly in half of the transgenic lines (Shen et al., 2018). Although previous studies have shown that the heterologous expression of *PeCPD* in wild-type *Arabidopsis* did not enhance the levels of BRs (Si et al., 2016).

### 5.3 Molecular mechanism of potato salt tolerance by BR signaling transduction

The levels of BRs changed, and the BR signaling process was elevated in the transgenic potatoes. GO analysis found that most of the DEGs were related to metabolic and cellular processes in biological processes. GO analysis was performed on the DEGs obtained under the same time salt stress treatment for both NT and T potato materials. As can be seen from **Supplementary Figure 1**, the three comparison groups (i.e., NT_0_vs_T_0, NT_24_vs_T_24, and NT_48_vs_T_48) were jointly enriched to biological processes of photosynthesis, light reaction, light harvesting, and generation of precursor metabolites and energy. The cellular components that were jointly enriched to the biological processes in the three comparison groups were thylakoid, photosynthetic membrane, and thylakoid membrane and photosystem. The molecular functions enriched by the three groups were chlorophyll-binding and carbon-carbon lyase activity. The DEGs between transgenic and NT plants were highly enriched in signaling pathways responding to salt stress. Significantly, the BR signaling process was elevated in the transgenic lines. The transcriptional levels of the signal transduction-related genes *BZR1*, *BRI1*, and *BAK1* increased in the transgenic lines. Transcript levels of the BR signaling pathway were also reported to be enhanced in transgenic lines of poplar and *Arabidopsis* (Si et al., 2016; Shen et al., 2018). Their RFLs were significantly higher than those of NT plants, and similar results were found in potatoes under saline conditions (Wambua et al., 2017).

**Figure 13:**
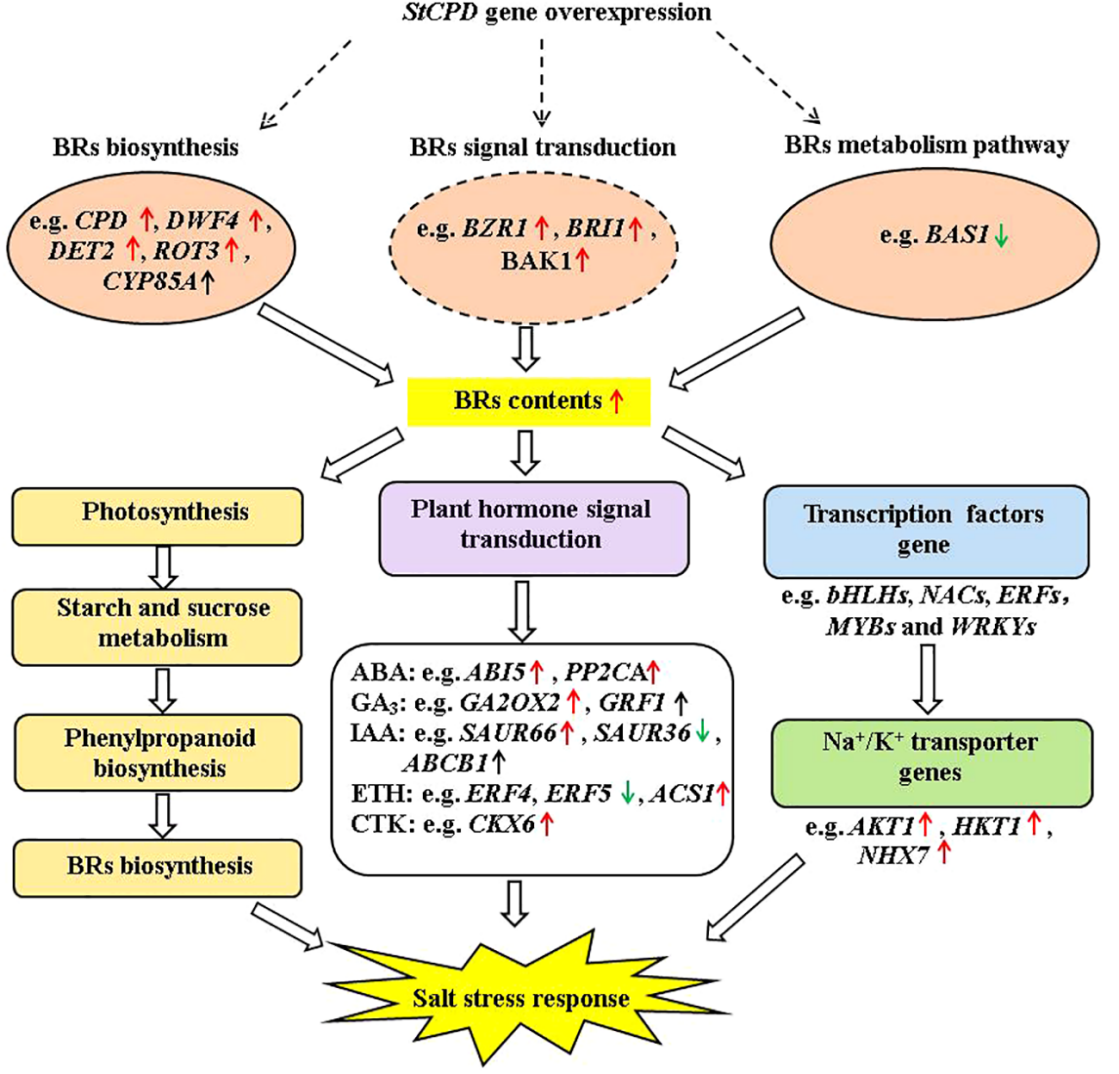
Diagram of a proposed model for regulation of the *StCPD* gene under salt stress tolerance in transgenic potato.

To mediate salt stress, three DEGs of *ROT3*, *ABI5*, and *PP2CA* were upregulated in both transgenic lines and NT plants at different treatment times in this study (**Figures 13A–F**). Transgenic lines had higher EBL contents than NT plants under salt stress. Therefore, the result suggested that the expression levels of synthesis genes *DWF4*, *DET2*, and *ROT3* increased whereas that of *CYP85A1* verified insignificantly in *StCPD* transgenic plants compared with those in the NT plants (**Figure 13A**).

Maintaining ion balance in cells is critical for their survival under salt stress. Leaf *NHX* transports excessive Na^+^ in the cytoplasmic matrix into the vacuole, thus reducing concentrations of Na^+^ in the cytoplasm (Zhu, 2016; Zhang et al., 2017). In this study, *HKT1* and *AKT1* were upregulated at 48-h salt treatment of transgenic and NT plants, suggesting that these genes reduced ion concentrations in the cytosol by transporting the absorbed excess Na^+^ into the vacuole, thus maintaining ion homeostasis and improving salt tolerance. Studies have shown that overexpression of the *GhNHX3D* in cotton plants could enhance salt resistance (Feng et al., 2021a); we found the *NHX7* exhibited identical expression patterns in potatoes at 48 h of salt stress. It was downregulated in NT and upregulated in transgenic lines, indicating that *NHX7* may be one of the key factors for the strong salt tolerance of transgenic plants.

As a high-affinity K^+^ transporter protein, *HKT* was related to Na^+^ uptake and transportation and mainly functioned as a Na^+^ transporter or Na^+^-K^+^ cotransporter (Wang et al., 2015; Zhang et al., 2017). As clarified here and by others (Han et al., 2018; Cao et al., 2019; Huang et al., 2019), it is generally accepted that salt stress was able to induce the expression of *HKT1* significantly. The upregulated expression levels in transgenic plants were greater than NT. The central roles of *AKT* were regulating the assimilation and transfer of K^+^ and promoting the selective uptake capacity of K^+^, thereby promoting the cellular ion balance of plants under salinity stress (Ma et al., 2014). The expression pattern of *AKT1* changed with treatment time and was consistent in all materials. Their expression was downregulated at 24 h and upregulated at 48 h. In the present study, the expression levels of the *AKT1* gene in transgenic plants were higher than those of NT, indicating that the former may reduce Na^+^ content through the Na^+^ transporter or Na^+^-K^+^ cotransporter, thus reducing maintenance of the intracellular Na^+^/K^+^ balance. *HKT1* and *AKT1* genes were upregulated in transgenic plants under salt stress, and the *AKT1* gene was downregulated and then upregulated in NT; the *HKT1* gene was upregulated then downregulated in NT, which indicated that transgenic plants materials could regulate cellular K^+^ uptake-related genes under salt stress, thus maintaining the intracellular Na^+^/K^+^ balance. However, *NHX7* and *AKT1* genes did not change obviously in transgenic and NT plants under severe salt stress (48 h treatment). In barley, salinity leads to an increase in expression levels of *AKT* (Gamal et al., 2021). In summary, salt-tolerant materials maintain cellular Na^+^/K^+^ homeostasis balance through ion transport-related genes, thereby reducing ion toxicity to resist salt stress (Zhang et al., 2020).

### 5.4 A possible salt-responsive molecular network in transgenic potatoes

In this study, a salinity-responsive molecular network was constructed in transgenic plants according to the results of phenotype, physiology and biochemistry, hormone, and transcriptome analysis (**Figure 13**). This network consisted of three fundamental functional parts: firstly, transgenic plants enhanced BR contents by upregulation of *DWF4*, *DET2, BZR1*, *BRI1*, and *BAK1* under salt stress. These upregulated protein kinase genes bind to TFs (transcription factors) to activate the expression of downstream salt-tolerant genes. In addition, salinity induced up- or down-expression of genes involved in photosynthesis, carbohydrate metabolism, and phenylpropanoid biosynthesis, which enhances the accumulation of primary and secondary metabolites, including BRs. Finally, those activating genes lead to changes in plant hormone signal transduction, improving plant hormone biosynthesis. These results indicated that the production of plant hormones, antioxidant enzymes, and secondary metabolites might have promoting effects on salt tolerance.

## 6 Conclusions

This study showed that *CPD* overexpression in the potato increased the contents of EBL, causing phenotypic and molecular changes in potatoes. These variations included enhancement in plant height, stem thickness, root length, fresh weight and size, changes of DEGs linked with salt stress, and DEGs linked with Na^+^/K^+^ transporter gene expression. The feedback of the potato plantlets to salt treatment varied depending on the treatment time. Our results indicated that transgenic plants were more tolerant to severe salt stress than NT. BR response to salt stress in transgenic plants was adjusted by expression levels of genes involved in BR biosynthesis, metabolism, and signaling process, which were related to Na^+^/K^+^ transporter, photosynthetic pigment, and osmotic compound. These results suggested the mechanisms participated in salinity tolerance in potatoes. Taken together, it may be concluded that BRs could relieve salt stress by changing the expression levels of the salt stress-related genes in the BR synthesis and signal transduction of potato plantlets.

## Data availability statement

The datasets presented in this study can be found in online repositories. The names of the repository/repositories and accession number(s) can be found below: PRJNA995223, https://www.ncbi.nlm.nih.gov/.

## Author contributions

XZ: Funding acquisition, Project administration, Supervision, Writing – original draft, Resources, Conceptualization. YM: Data curation, Formal analysis, Writing – review & editing, Visualization. RM: Software, Writing – review & editing, Formal analysis, Validation. CL: Software, Writing – review & editing, Validation. ZL: Investigation, Writing – review & editing, Validation. DZ: Writing – review & editing, Methodology, Supervision. SC: Investigation, Writing – review & editing, Supervision. JL: Writing – review & editing, Resources, Methodology. WT: Writing – review & editing, Resources, Methodology.
