# Supplementary material for: Physiological responses and transcriptomic analysis of StCPD gene overexpression in potato under salt stresses
Source: Front Plant Sci. 2024 Feb 16;15:1297812. doi: 10.3389/fpls.2024.1297812 (PMC10906663; doi:10.3389/fpls.2024.1297812)
Supplement: Supplementary file 1 [file DataSheet_1.pdf]

Supplementary Material

1 Supplementary Figures and Tables

1.1 Supplementary Figures

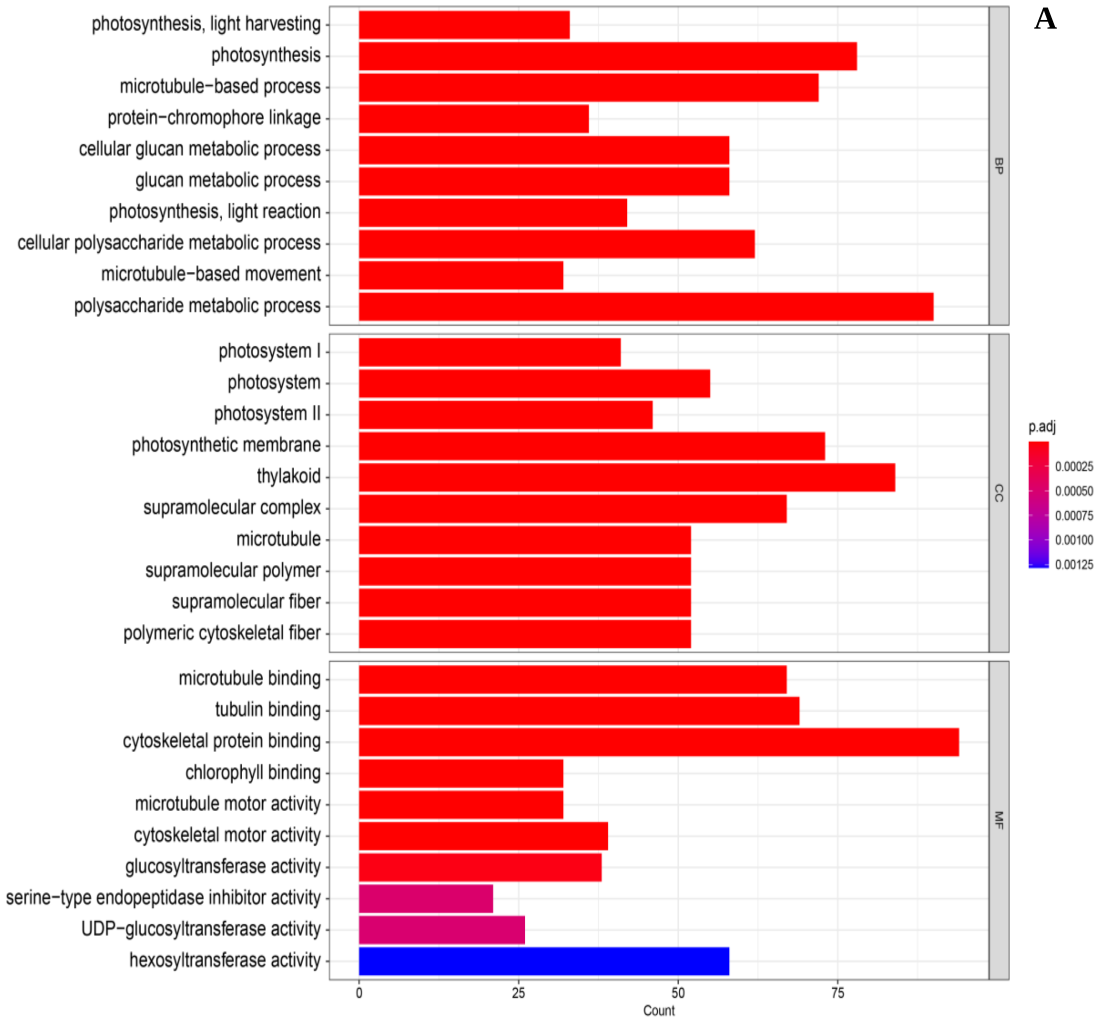

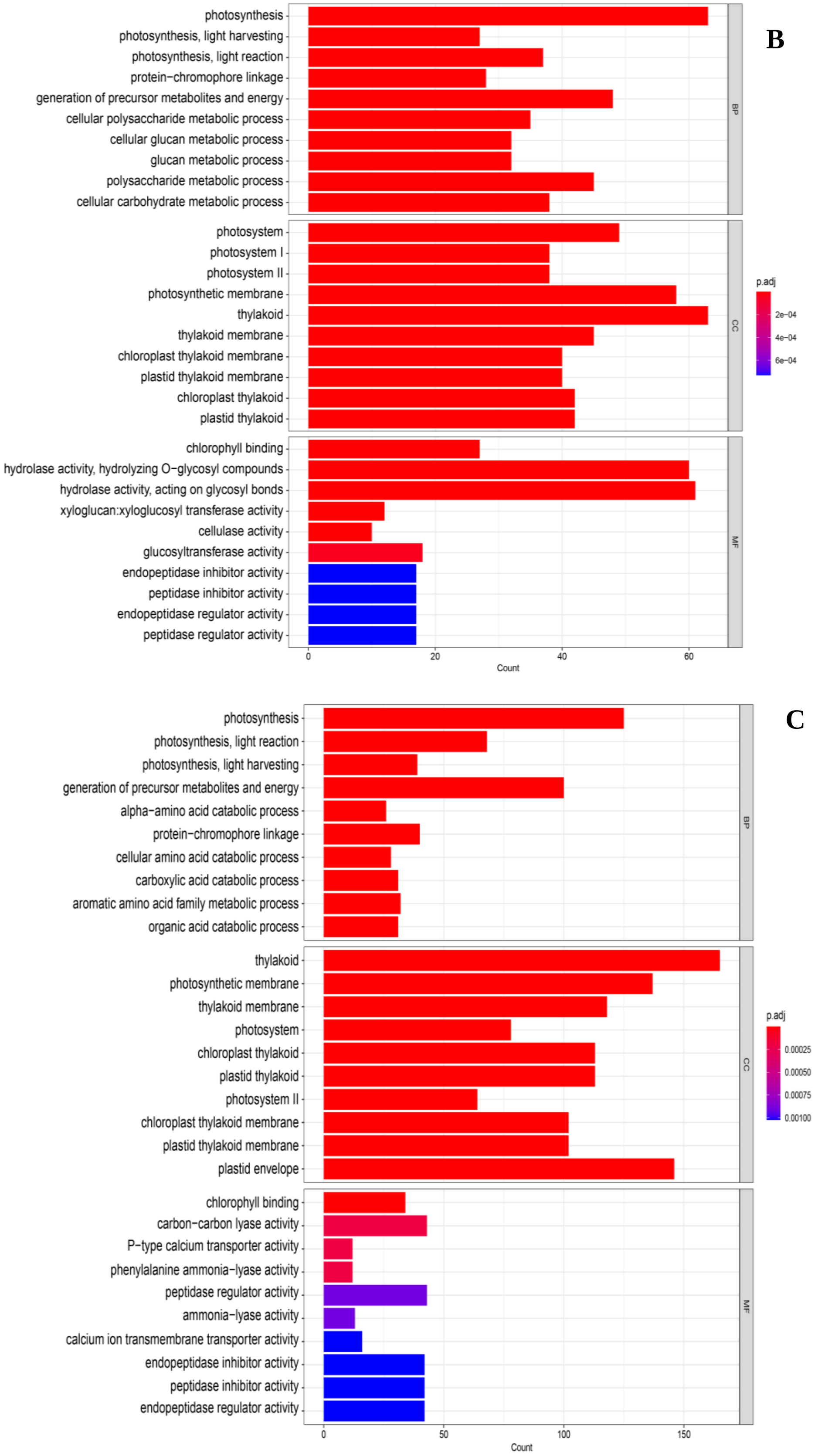

Figure S1. GO annotation of DEGs in NT (A), T (B) and NT vs T (C) potatoes under salt stress

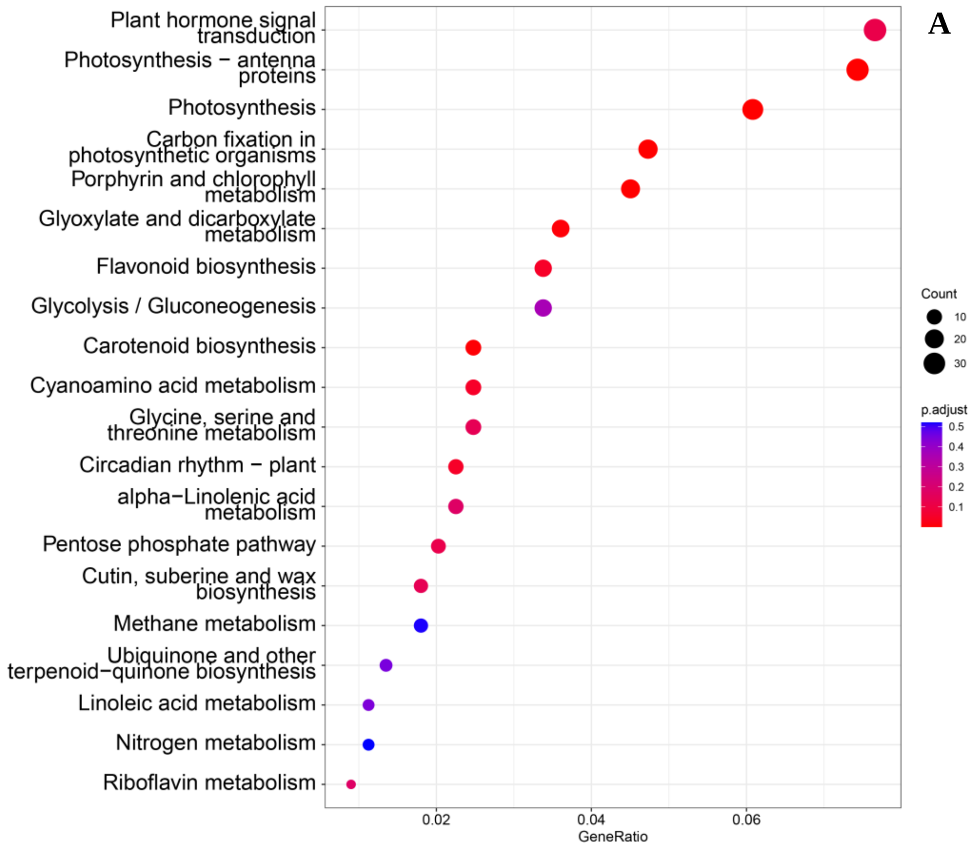
A
Plant hormone signal transduction
Photosynthesis − antenna proteins
Photosynthesis
Carbon fixation in photosynthetic organisms
Porphyrin and chlorophyll metabolism
Glyoxylate and dicarboxylate metabolism
Flavonoid biosynthesis
Glycolysis / Gluconeogenesis
Carotenoid biosynthesis
Cyanoamino acid metabolism
Glycine, serine and threonine metabolism
Circadian rhythm − plant
alpha−Linolenic acid metabolism
Pentose phosphate pathway
Cutin, suberine and wax biosynthesis
Methane metabolism
Ubiquinone and other terpenoid−quinone biosynthesis
Linoleic acid metabolism
Nitrogen metabolism
Riboflavin metabolism
0.02
0.04
0.06
GeneRatio
Count
10
20
30
p.adjust
0.5
0.4
0.3
0.2
0.1

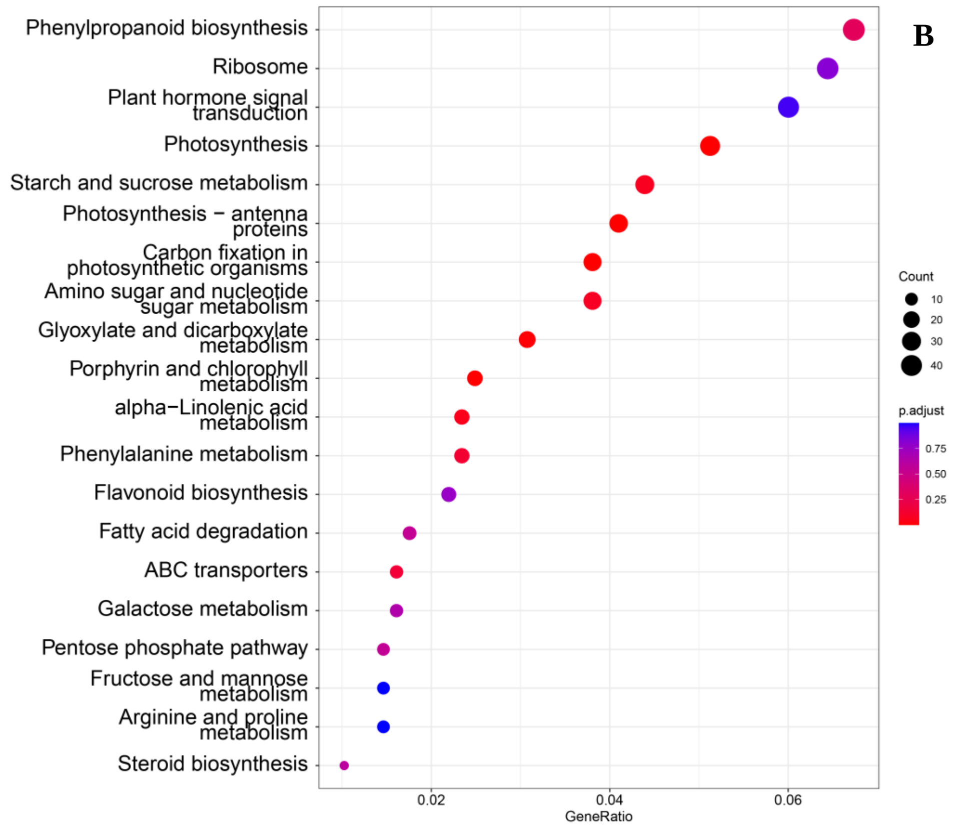
B
Phenylpropanoid biosynthesis
Ribosome
Plant hormone signal transduction
Photosynthesis
Starch and sucrose metabolism
Photosynthesis − antenna proteins
Carbon fixation in photosynthetic organisms
Amino sugar and nucleotide sugar metabolism
Glyoxylate and dicarboxylate metabolism
Porphyrin and chlorophyll metabolism
alpha−Linolenic acid metabolism
Phenylalanine metabolism
Flavonoid biosynthesis
Fatty acid degradation
ABC transporters
Galactose metabolism
Pentose phosphate pathway
Fructose and mannose metabolism
Arginine and proline metabolism
Steroid biosynthesis
0.02
0.04
0.06
GeneRatio
Count
10
20
30
40
p.adjust
0.75
0.50
0.25

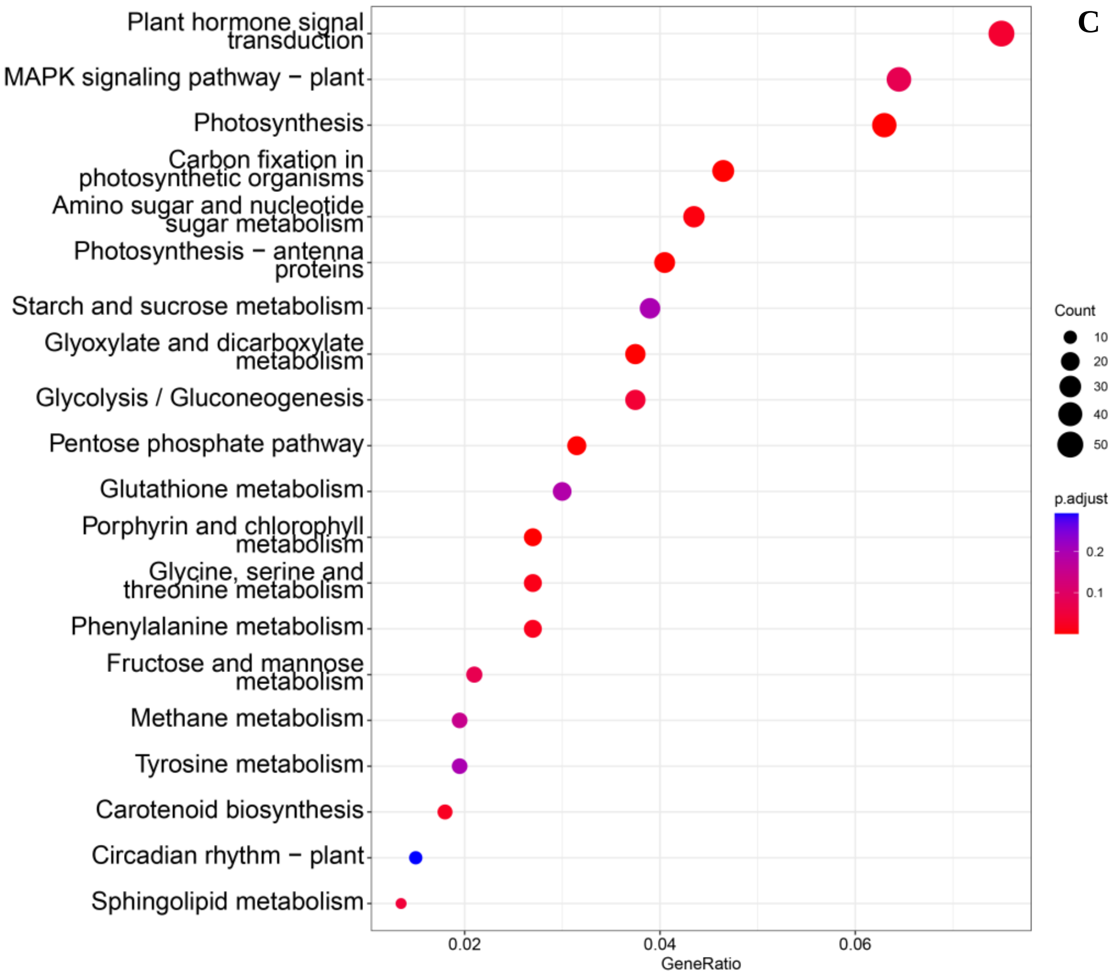

Figure S2. KEGG pathway analysis of NT and T potato DEGs under salt stress
A. NT_0_vs_T_0; B. NT_24_vs_T_24; C. NT_48_vs_T_48

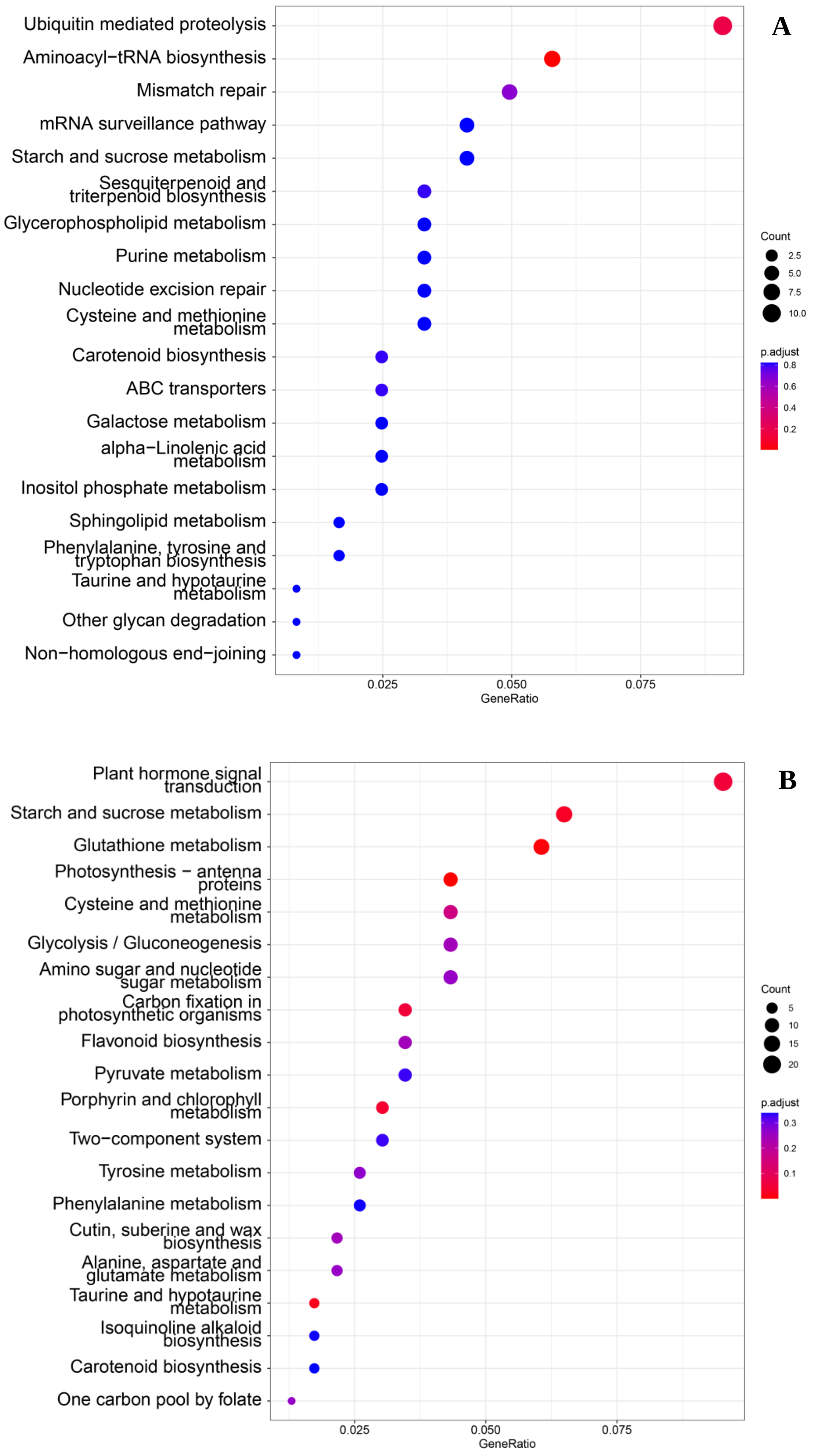

Figure S3. KEGG pathway analysis of NT potato DEGs under salt stress

A. NT_0_vs_NT_24; B. NT_0_vs_NT_48

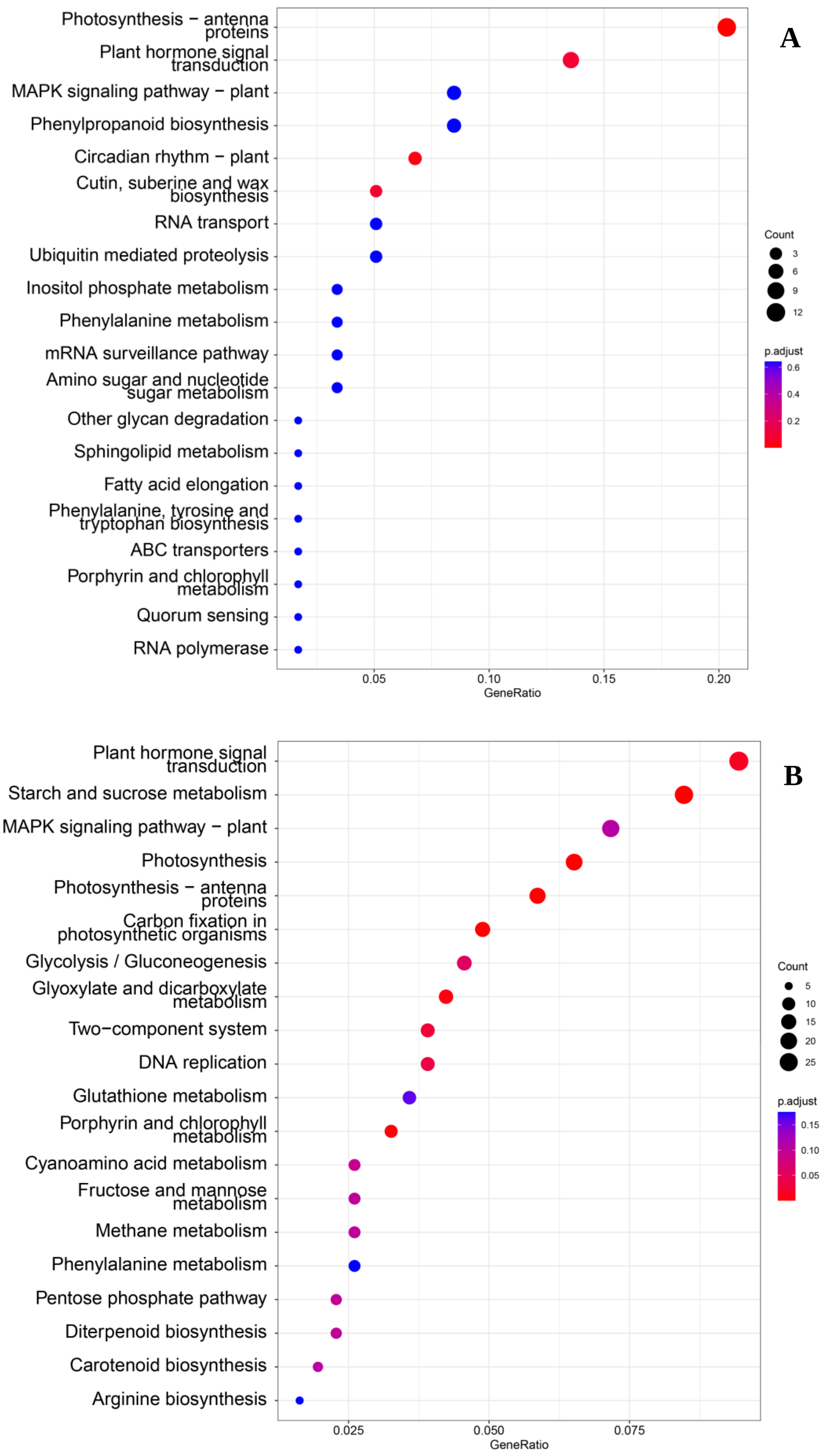

Figure S4. KEGG pathway analysis of T potato DEGs under salt stress

A. T_0_vs_T_24; B. T_0_vs_T_48

1.2 Supplementary Tables

Table S1 Sequences of primer employed in the qRT-PCR analysis

Gene name	Primer sequences (5' to 3')
StEF-1α	Forward: ATTGGAAACGGATATGCTCCA<br>Reverse: TCCTTACCTGAACGCCTGTCA
DET2	Forward: TATCCTCTTCCCCTTCGGCA<br>Reverse: CGACCCACCCTTTGAGTTGA
CYP85A1	Forward: TTCTGAATGAAGACCGGCCC<br>Reverse: GCCACATACCCTGACTGCTA
BZR1	Forward: AACAGTGCCCCTGTAACTCC<br>Reverse: AAACGCTGACCCCTAGTTGG
BRI1	Forward: CCTTGTCCCTCTTTTGGGCT<br>Reverse: TGGTGTAGGAAAGCCAACCC
GRF1	Forward: GTGGTGGGGGCTGATGTTAG<br>Reverse: ATGCTGCTTGCTGACAAACC
ROT3	Forward: AAAATCGCCGCAACTCAAGG<br>Reverse: TCCAGGGTCCACGCTTAGTA
BAS1	Forward: AGGTCGCAGTTTACCCTCCT<br>Reverse: AACAAAGTGACCTCTTACTGCT
CPD	Forward: GCGTAAAAATCGACTCCCGC<br>Reverse: GGGTGGTGAAAATGTTGCCG
DWF4	Forward: TGACCGACCTCACGACTTTG<br>Reverse: TTGGCCAGTTCAGATCCTGC
HKT1	Forward: GGCCATTGCAAGGATGCTAC<br>Reverse: ACTTTCCAAGCCTTTCCACCT
NHX7	Forward: CCAAGCGCACCAATTGAACA<br>Reverse: GGAAAGCTTGGTCTTCCCGA
AKT1	Forward: TGCTAGCTCGCGGTAGAATG<br>Reverse: TGGATCAAGACCGCGTTTCA
CKX6	Forward: TTTCGTCACGGCCCTCAAAT<br>Reverse: TTTCGGGGCTCTTTCGAGTG
BAK1	Forward: TTGTCAATGGTCGCCGGTAA<br>Reverse: AGGGTTGCATCCCAACTCTG
ERF5	Forward: AAGGCATTTTTGAGGTGCCG<br>Reverse: CGTAGACACACCCACGATCT
ACS1	Forward: ATTTGCACTGCTGAAGGAGC<br>Reverse: CAGCCAAGCAAAAAGCAAGC
ERF4	Forward: GTTGAATCGTCGAGTCCGGT<br>Reverse: ACCTCCACCAGAGATCGGAA
GA2OX2	Forward: CGGATGACAAGTCCATTGCG<br>Reverse: GAGACGGGACGCGAAAAGAA
ABI5	Forward: TCGCGATTCCTGGTTATCCG<br>Reverse: ACTACTTGCCCGTAGCCAAC
PP2CA	Forward: GTTCTCGGCGTTTTGGCAAT<br>Reverse: CGTTCGATACCACGTCCCAT
SAUR36	Forward: GGAAACAAGCGGCTGAGTTG<br>Reverse: ATCAATCGGTTCTTGCCCGA
SAUR66	Forward: CAAGCACGCTTTGTCATTCCA<br>Reverse: TAATAGGGCCACCACTCGGA
ABCB1	Forward: TTCGTATTGCAGTGGCGTCT<br>Reverse: ATGAGCAGCTTCCAAGTCCC

Table S2 Summary of sequencing data of transcriptome

Sample	Raw_reads	Clean_reads	Clean_bases	Q20 (%)	Q30 (%)	GC (%)
NT_0_1	53,333,332	53,333,300	7.85	97.89	93.88	43.46
NT_0_2	51,927,086	51,927,054	7.72	97.96	94.01	42.08
NT_0_3	53,333,332	53,333,304	7.87	97.68	93.32	43.17
NT_24_1	53,333,332	53,333,322	7.82	97.7	93.36	42.85
NT_24_2	53,333,332	53,333,318	7.7	97.98	94.06	42.61
NT_24_3	53,333,332	53,333,324	7.9	97.84	93.72	42.41
NT_48_1	53,333,332	53,333,302	7.92	97.42	92.56	44.66
NT_48_2	53,333,332	53,333,266	7.87	97.8	93.63	42.92
NT_48_3	53,333,332	53,333,320	7.93	97.3	92.37	42.7
T_0_1	40,071,212	40,071,188	5.94	97.93	93.89	43.04
T_0_2	53,333,332	53,333,292	7.45	98.22	94.56	42.8
T_0_3	53,333,332	53,333,324	7.96	97.56	92.95	42.94
T_24_1	49,746,924	49,746,892	7.42	97.69	93.28	43.74
T_24_2	53,333,332	53,333,324	7.93	97.74	93.44	42.65
T_24_3	53,333,332	53,333,300	7.77	97.89	93.84	43.34
T_48_1	53,333,332	53,333,280	7.88	97.44	92.59	43.78
T_48_2	53,333,332	53,333,314	7.95	97.54	93	42.65
T_48_3	53,333,332	53,333,316	7.95	97.79	93.54	42.6

Note: Raw_ Reads refers to the number of dual ended Reads in raw data, in pairs; Clean_ Reads refers to the number of dual ended Reads in Clean Data, in pairs; Clean_ Bases (G) refers to the total data amount of Reads in Clean Data, G; Q20 (%) refers to the proportion of bases with a Q value greater than 20 among all bases, %; Q30 (%): refers to the proportion of bases with a Q value greater than 30 among all bases; GC (%): The percentage of the number of bases G and C in the total base, %.

Table S3 Functional annotation for all unigenes

All Unigenes	Annotation genes	KEGG	GO	NR	Swissprot	TrEMBL	eggNOG	Pfam	TF_Family
32,917	32,372(98.34%)	7466(22.68%)	15045(45.71%)	31931(97.00%)	24273(73.74%)	32291(98.10%)	31683(96.25%)	28751(87.34%)	18695(56.79%)

Table S4 FPKM values and RELs of hormone and osmosis-related DEGs in NT and T potatoes responding to salt stress

Related metabolic pathways	Gene name	Gene ID	FPKM values			RELs		
			T_0_vs_NT_0	T_24_vs_NT_24	T_48_vs_NT_48	T_0_vs_NT_0	T_24_vs_NT_24	T_48_vs_NT_48
Brassinosteroid	*DWF4*	Soltu.DM.02G025220	1.96	1.46	0.92	4.55	1.07	0.91
Brassinosteroid	*CYP85A1*	Soltu.DM.02G030630	0.93	0.79	1.26	0.40	0.57	1.17
Brassinosteroid	*BZR1*	Soltu.DM.12G005470	1.78	2.31	1.11	2.35	5.90	2.41
Brassinosteroid	*ROT3*	Soltu.DM.02G024550	1.06	0.79	1.16	1.97	1.27	1.48
Brassinosteroid	*BAS1*	Soltu.DM.12G024720	0.60	0.50	0.67	0.68	1.22	2.57
Brassinosteroid	*DET2*	Soltu.DM.09G000750	0.97	0.78	1.31	1.99	1.09	1.12
Brassinosteroid	*BRI1*	Soltu.DM.04G023990	1.05	1.63	0.74	2.46	5.77	1.18
Brassinosteroid	*BAK1*	Soltu.DM.10G012540	1.41	1.19	0.97	1.36	2.71	2.33
Ethylene	*ERF5*	Soltu.DM.03G014580	0.64	1.62	1.02	0.39	8.03	1.10
Ethylene	*ACS1*	Soltu.DM.08G028290	2.67	5.14	1.10	1.13	2.28	0.94
Ethylene	*ERF4*	Soltu.DM.10G005000	1.08	3.86	1.04	1.02	9.30	1.40
GA	*GA2OX2*	Soltu.DM.01G025170	23.03	25.79	2.80	3.50	3.82	1.38
GA	*GRF1*	Soltu.DM.07G012510	1.04	2.09	7.92	0.89	6.79	8.99
Cytokinin	*CKX6*	Soltu.DM.01G026480	1.90	4.64	1.11	1.32	5.36	1.75
ABA	*ABI5*	Soltu.DM.09G003620	11.67	5.80	4.11	2.80	1.51	1.24
ABA	*PP2CA*	Soltu.DM.05G023010	1.53	1.27	1.23	1.55	1.36	1.55
IAA	*SAUR36*	Soltu.DM.08G025440	0.46	1.61	1.58	0.45	2.15	0.44
IAA	*SAUR66*	Soltu.DM.11G001370	1.53	0.89	1.10	2.39	1.82	2.64
IAA	*ABCB1*	Soltu.DM.09G005360	0.93	1.19	0.67	0.92	1.24	0.85
Na^+/ K^+ Transporter Gene	*HKT1*	Soltu.DM.07G005700	0.50	0.34	1.49	0.99	1.04	8.23
Na^+/ K^+ Transporter Gene	*NHX7*	Soltu.DM.01G000050	0.90	1.24	1.29	0.88	310.53	551.69
Na^+/ K^+ Transporter Gene	*AKT1*	Soltu.DM.12G024710	1.69	1.65	1.11	1.48	2.17	1.30

Table S5 log_2FC of hormone and osmosis-related DEGs in NT and T potatoes responding to salt stress

Related metabolic pathways	Gene name	Gene ID	log_2FC			
			NT_0_vs_NT_24	NT_0_vs_NT_48	T_0_vs_T_24	T_0_vs_T_48
Brassinosteroid	*CPD*	Soltu.DM.06G009120	-0.77	0.25	0.47	-0.35
Brassinosteroid	*DWF4*	Soltu.DM.02G025220	-0.16	0.51	-0.38	0.02
Brassinosteroid	*CYP85A1*	Soltu.DM.02G030630	0.05	-0.21	0.12	0.31
Brassinosteroid	*BZR1*	Soltu.DM.12G005470	-0.37	0.15	0.1	-0.36
Brassinosteroid	*ROT3*	Soltu.DM.02G024550	0.49	0.27	0.21	0.77
Brassinosteroid	*BAS1*	Soltu.DM.12G024720	-0.32	-0.02	-0.81	-0.07
Brassinosteroid	*DET2*	Soltu.DM.09G000750	-0.04	-0.56	0.06	-0.02
Brassinosteroid	*BRI1*	Soltu.DM.04G023990	-0.24	0.42	0.27	-0.33
Brassinosteroid	*BAK1*	Soltu.DM.10G012540	0.05	0.32	-0.19	-0.11
Ethylene	*ERF5*	Soltu.DM.03G014580	-1.08	-0.68	0.34	0.27
Ethylene	*ACS1*	Soltu.DM.08G028290	-0.04	0.44	1.16	-0.16
Ethylene	*ERF4*	Soltu.DM.10G005000	-1.04	-0.05	0.78	0.23
GA	*GA2OX2*	Soltu.DM.01G025170	-0.23	2.3	0.39	0.27
GA	*GRF1*	Soltu.DM.07G012510	-0.33	0.24	0.69	-0.99
Cytokinin	*CKX6*	Soltu.DM.01G026480	-1.08	0.86	0.11	0.12
ABA	*ABI5*	Soltu.DM.09G003620	1.28	2.17	-0.21	0.05
ABA	*PP2CA*	Soltu.DM.05G023010	0.67	0.51	0.42	0.27
IAA	*SAUR36*	Soltu.DM.08G025440	-0.56	-0.5	1.32	0.93
IAA	*SAUR66*	Soltu.DM.11G001370	0	0.39	-0.73	0.14
IAA	*ABCB1*	Soltu.DM.09G005360	-0.22	0.09	0.05	-0.59
Na^+/ K^+ Transporter Gene	*HKT1*	Soltu.DM.07G005700	0.75	0.25	0.2	1.76
Na^+/ K^+ Transporter Gene	*NHX7*	Soltu.DM.01G000050	-0.32	-0.1	0.04	0.31
Na^+/ K^+ Transporter Gene	*AKT1*	Soltu.DM.12G024710	-0.12	0.3	-0.19	-0.07
